# Nutraceutical Compounds Targeting Inflammasomes in Human Diseases

**DOI:** 10.3390/ijms21144829

**Published:** 2020-07-08

**Authors:** Beatriz Castejón-Vega, Francesca Giampieri, José M. Alvarez-Suarez

**Affiliations:** 1 Research Laboratory, Oral Medicine Department, University of Sevilla, 41009 Sevilla, Spain; beacastej92@outlook.es; 2 Nutrition and Food Science Group, Department of Analytical and Food Chemistry, CITACA, CACTI, University of Vigo, 36310 Vigo, Spain; f.giampieri@univpm.it; 3 Dipartimento di Scienze Cliniche Specialistiche ed Odontostomatologiche (DISCO)-Sez, Biochimica, Facoltà di Medicina, Università Politecnica delle Marche, 60131 Ancona, Italy; 4 College of Food Science and Technology, Northwest University, Xi’an 710069, China; 5 Facultad de Ingeniería y Ciencias Aplicadas (FICA), AgroScience & Food Research Group, Universidad de Las Américas, 170125 Quito, Ecuador; 6 King Fahd Medical Research Center, King Abdulaziz University, 21589 Jeddah, Saudi Arabia

**Keywords:** NLRP3 inflammasomes, nutraceuticals, immune system, health, cardiovascular diseases, type 2 diabetes, cancer, neurological diseases

## Abstract

The macromolecular complex known as “inflammasome” is defined as an intracellular multi-protein complex composed of a sensor receptor (PRR), an adaptor protein and an effector enzyme (caspase-1), which oligomerize when they sense danger, such as how the NLR family, AIM-2 and RIG-1 receptors protect the body against danger via cytokine secretion. Within the NLR members, NLRP3 is the most widely known and studied inflammasome and has been linked to many diseases. Nowadays, people’s interest in their lifestyles and nutritional habits is increasing, mainly due to the large number of diseases that seem to be related to both. The term “nutraceutical” has recently emerged as a hybrid term between “nutrition” and “pharmacological” and it refers to a wide range of bioactive compounds contained in food with relevant effects on human health. The relationship between these compounds and diseases based on inflammatory processes has been widely exposed and the compounds stand out as an alternative to the pathological consequences that inflammatory processes may have, beyond their defense and repair action. Against this backdrop, here we review the results of studies using several nutraceutical compounds in common diseases associated with the inflammation and activation of the NLRP3 inflammasomes complex. In general, it was found that there is a wide range of nutraceuticals with effects through different molecular pathways that affect the activation of the inflammasome complex, with positive effects mainly in cardiovascular, neurological diseases, cancer and type 2 diabetes.

## 1. Background

The immune system (IS) is a host’s defense system that is associated with many biological structures, such as organs, cells, proteins and processes that protect the body against diseases. Vertebrates have two types of IS: innate and adaptive. The innate immune system is activated first and it is considered a sophisticated system that senses danger signs, including pathogenic microbes or host-derived signals of cellular stress. Furthermore, it initiates the inflammatory response by secreting cytokines and chemokines, which play a very important role in protecting the body against pathogens and damaged cells [1,2].

The innate immune system is not specific to a particular pathogen; it senses evolutionary conserved structures on it, which are known as pathogen-associated molecular patterns (PAMPs). It also senses damage-associated molecular patterns (DAMPs), which are a set of host-derived molecules that signal cellular stress, damage and death. These structures are identified via sensor receptors called pattern recognition receptors (PRRs), which are expressed by cells at the front line of defense against infection. These cells can be hematopoietic or non-hematopoietic and include dendritic cells, macrophages and epithelial cells. Because PAMPs are broadly expressed in pathogens but not in host cells, PRRs discriminate between self and non-self [3,4].

**Figure 1:**
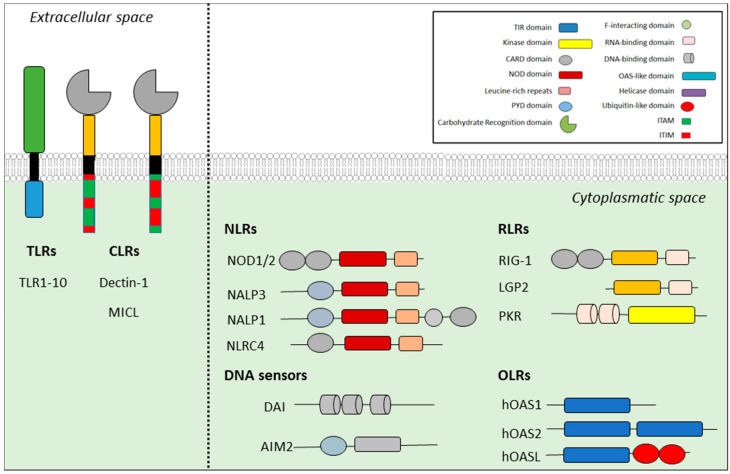
Cellular pattern recognition receptors (PRRs). Toll-like receptors (TLRs) are transmembrane receptors expressed in cellular and endosomal membranes, which are comprised of 10 members in human beings. They each recognize distinct pathogen-associated molecular patterns (PAMPS) derived from various microbial pathogens, such as viruses, fungi, bacteria and protozoa. TLRs are detected via the LRRdomain and a signal is sent through intracellular space via the TIR domain. Retinoic acid inducible gene-I (RIG1)-like receptors (RLRs) detect RNA and activate the helicase domain or the kinase domain. There are two intracellular detectors of DNA called DAI and AIM2. Nucleotide-binding-and-oligomerization-domain (NOD)-like receptors (NLRs) belong to a very large family of intracellular PRRs, whilst c-type lectin receptors (CLRs) belong to a large family of proteins, which play an essential role in antifungal immunity. Only a few CLRs function as PRRs, for instance Dectin-1 and MICL, by recognizing carbohydrate ligands from pathogens. Signals are transmitted through the N-terminal domain.

A few families of PRRs exist to respond to pathogens and harmful particles and they can be divided into two groups based on their cellular location, where they can be transmembrane receptors and those located in intracellular compartments, or into six groups based on common structures and functional domains (Figure 1). Firstly, transmembrane PRRs scan the extracellular space and endosomal compartments and include the families of toll-like receptors (TLRs) and c-type lectin receptors (CLRs). Secondly, intracellular PRRs include the nucleotide-binding-and-oligomerization-domain (NOD)-like receptors (NLRs), absent-in-melanoma (AIM)-like receptors (ALRs), retinoic acid inducible gene-I (RIG1)-like receptors (RLRs) and olygoadenylate synthetase-like receptors (OLRs) [1,2,5,6]. Due to the quantity and location of PRRs, which bind a diverse array of targets including polysaccharides, lipoproteins, nucleic acid, carbohydrates and conserved microbial proteins, our bodies are well-protected against many dangers [5].

The adaptive immune system defense is antigen specific and generates memory that enhances the immune response to a consecutive infection with the same antigen. These more sophisticated mechanisms are activated by the innate IS and both work together to eliminate the pathogens. Adaptive immunity is carried out by lymphocytes B and T (white cells), which execute antibody responses and cell-mediated immune responses, respectively. Antibodies are secreted through the body and inactivate the pathogens by binding to them, which also makes the pathogens visible so that they can be destroyed by phagocytic cells. On the other hand, T-cells are involved in the recognition of infected cells and they eliminate them [7].

## 2. Inflammasomes

Many of the PRRs initiate inflammatory signaling; the signal transduction depends on the nature of the responding cells and the pathogen and, commonly, it includes the activation of NF_Ϗ_β and AP-1 transcription factors that drive the production of pro-inflammatory cytokines and chemokines. There are specific types of PRRs that oligomerize when they sense danger, such as the NLR family, AIM-2 and RIG-1 receptors, and these are called inflammasomes. They are defined as an intracellular multi-protein complex composed of a sensor receptor (PRR), an adaptor protein, and an effector enzyme (caspase-1). After the activation of caspase-1, the processing and maturation of pro-inflammatory cytokines (IL-1β and IL-18) takes place. This protects the body against danger via cytokine secretion; otherwise, the initiation of an inflammatory programmed cell death called pyroptosis can occur [2,8].

The NLR family consists of 22 genes, all of which are characterized by the presence of central nucleotide-binding and oligomerization domain (NATCH), responsible for the ATP-dependent oligomerization of the inflammasome. The domain is unique and common to all NLR family members. At the carboxyl terminus, there are leucine-rich repeats (LRRs) responsible for detecting the ligands (PAMPs and DAMPs), while at the N-terminus, there are caspase recruitment (CARD) or pyrin (PYR) domains, which through protein–protein interaction are both responsible for the pro-caspase-1 cleavage and therefore the secretion of pro-inflammatory cytokines. One of these protein interactions is with an adaptor protein: apoptosis-associated speck-like protein (ASC), which has a caspase-activating recruitment domain, provoking the cleavage of pro-caspase-1. Within the NLR members, NLRP3 is the most well-known and studied inflammasome and has been linked to many diseases: metabolic disorders such as type 2 diabetes [9], obesity, gout [10] and atherosclerosis [11,12,13,14]; diseases affecting the central nervous system such as Alzheimer´s [15,16] and Parkinson´s [17], cancer [18] and inflammatory diseases [19,20,21].

Meanwhile, the inflammasomes NLRP1, NLRC3, NLRC4, NALP10 and NLRP6 are involved in the inflammatory process and thus are implicated in many diseases and pathological states [1,4]. All of them are activated by different ligands: NLRP3 can be activated by a huge range of DAMPs, such as cholesterol crystals, ATP, ROS, hyaluronan, monosodium urate, low intracellular K^+^, aluminum hydroxide, amyloid polypeptide and high intracellular glucose, as well as PAMPs such as microbial components. NLRP1 is activated by anthrax (a lethal toxin) and NLRC4 by the flagellin of several bacteria [22].

In addition to the NLR family, AIM2 (Figure 1) is another well-known inflammasome and it is composed of a HIN-200 domain that senses bacterial and viral cytosolic-double stranded DNA (dsDNA) and a PYR domain, which makes an association with ASC in order to recruit pro-caspase-1 and start the inflammatory cascade [22].

## 3. Mechanisms of NLRP3 Activation

**Figure 2:**
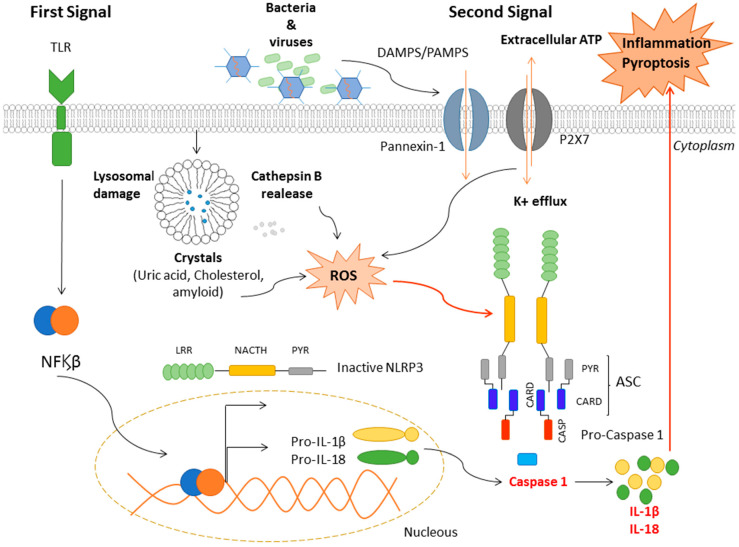
Mechanisms of NLRP3 activation. There are two consecutive signals that are needed in order to have an activated inflammasome. The first is responsible for the activation of the NF_Ϗ_β pathway and upregulating inactive pro-inflammatory cytokines such as IL-1β and IL-18, as well as transcribing NLRP3 proteins. The second signal carries out the recruitment of apoptosis-associated speck-like protein (ASC) and pro-caspase-1 and the NLRP3 oligomerization. Then, the pro-inflammatory cytokines are activated and released into extracellular space. Within the NLRP3 ligands, extracellular ATP, K^+^ efflux and Ca^+2^ trigger the inflammasome through the Pannexin-1, P2 × 7 and TRMP2 receptors. Exogenous particulate matter destabilizes and damages lysosomes and thus, numerous inner enzymes such as cathepsin B are released into the cytoplasm and activate the NLRP3. High levels of Reactive Oxygen Species (ROS) cause the dissociation of TNXIP from thioredoxin (TRX) and binding to NLRP3.

The mechanisms of NLRP3 activation (Figure 2) comprise two steps conducted by two activating signals. The first signal, also known as priming, is responsible for the activation of the NF_Ϗ_β pathway. It activates the transcriptional factor NF_Ϗ_β, which goes inside the nucleus and transcripts the pro-IL-1β and pro-IL-18 by binding itself to the DNA, as well as the production of mRNA of inactive NLRP3. Its signals are mediated by microbial ligands and endogenous cytokines that bind to the TLR and TNFR receptor, respectively.

The second signal is made by the ligands (PAMPS or DAMPs) that trigger NLRP3 and they are sensed by the LRR domain. In addition, NLRP3 senses Reactive Oxygen Species (ROS), which are mostly produced and activated by the inflammasomes activators. Then, the NATCH domain makes conformational changes in order to assemble the inflammasome structure and the recruitments of ASC and pro-caspase-1. Next, the proteolytic cleavage of the pro-caspase-1 converts the pro-IL-1β and pro-IL-18 into active cytokines that are secreted to the extracellular space and the inflammation cascade is initiated, which can lead to inflammation or pyroptosis. The protein that regulates thioredoxin (TRX) is thioredoxin-interacting protein (TXNIP) and it is an upstream NLRP3 activator. In high levels of ROS, TXNIP dissociates from TRX and binds and activates the NRLP3 inflammasome, which promotes IL-1β maturation and secretion [23].

## 4. Nutraceutical Compounds

Currently, more people are worried about their lifestyles and nutritional habits, because of the huge number of diseases that appear to be linked to the food we eat, such as type 2 diabetes, atherosclerosis, obesity, liver, kidney, bones diseases and cancer [24]. In addition, consumers are unhappy with the high prices of disease treatments, so searching for natural medicines to avoid an illness and/or to cure a disease is essential.

Food contains a huge variety of bioactive compounds with healthy benefits that help us protect ourselves from illnesses, and thus offer us an easy way to improve our quality of life, such as carotenoids, essential oils, vitamins, etc. Among their beneficial functions are anti-aging properties and protection against type 2 diabetes, cancer, cardiovascular and neurodegenerative diseases. Nowadays, there is growing evidence of health improvement by consuming these compounds with anti-inflammatory, antioxidant, antihypertensive and lipid-lowering properties [25,26]. However, their stability and therefore their properties may be lost during digestion and absorption in the body. Hence, these compounds are studied in order to become stabilized and increase their bioavailability [27].

Nutraceutical is a hybrid term between “nutrition” and “pharmacological” that was formulated to refer to a wide range of bioactive compounds contained in food. They can be organized by their food source, such as dietary fiber, probiotics, prebiotics, polyunsaturated fatty acids, antioxidants, vitamins, polyphenols and spices. There is a clear difference between functional food and nutraceuticals. Functional food items not only supply us with basic nutritional properties, they also contain an extensive array of phytochemicals and biologically active components with physiological benefits that potentially improve health, reducing the risk of suffering from diseases. Nonetheless, nutraceuticals are considered a concentrated form of the bioactive compounds found in foods that are presented in non-food matrices such as pills, extracts, powders and tablets [28].

**Figure 3:**
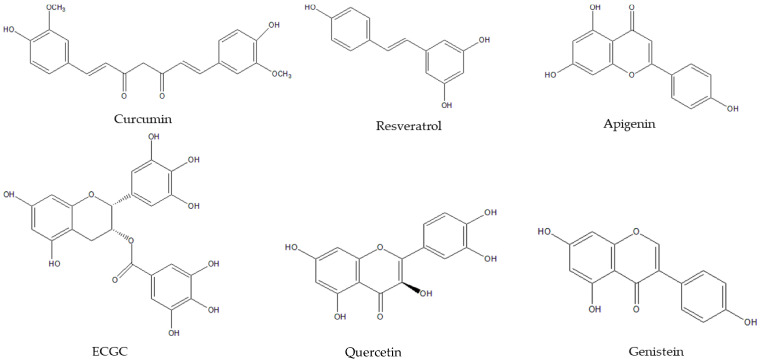
Structure of some of the most common polyphenolic compounds. They contain one or more benzene rings joined to hydroxyl groups.

The most important and abundant phytochemical compounds in the human diet are polyphenols, whose structure is composed by one or more benzene rings joined to hydroxyl groups, which confers them with their antioxidant capacity. In addition, polyphenols are classified in different subclasses, such as flavonoids (the widest group), phenolic acids, phenolic alcohols, stilbenes and lignans. Figure 3 displays some of the most studied nutraceuticals, which are polyphenols.

With this background, here we present a review about the relationship between different common diseases, such as cardiovascular and neurological diseases, type 2 diabetes and cancer, which are associated with high levels of inflammation, with the activation of inflammasomes (essentially NLRP3) and the subsequent use of several nutraceutical compounds to target them.

## 5. Nutraceutical Compounds, the NLRP3 Inflammasome and Cardiovascular Diseases

Inflammasomes occupy a central role in the development of cardiovascular diseases (CVDs), as they are involved in the development of arteriosclerotic problems [29]. CVDs are strongly associated with glucose metabolism disorders including type 2 diabetes. It is documented that two thirds of diabetic patients die from cardiovascular disease, in which endothelial senescence is its early manifestation. For this reason, anti-senescence drugs can be used to treat diabetes complications and age-related vascular diseases. Although many stimuli can promote cellular senescence, all of them induce inflammation and thus, the intake of flavonoids [30], alkaloids [31], iridoids [32], and polyunsaturated fatty acids (PUFAs) [33], could interfere with the activation of the NLRP3 inflammasome.

After the endothelial senescence, NLRP3 is activated by many types of stimuli, in this case by intracellular cholesterol crystals. The latter are one of the main inductors of ROS productions, which are ligands for the activation of NLRP3. This causes endothelial senescence and therefore endothelial dysfunction, which initiates vascular injury. Once the downstream effectors of the NLRP3 pathway are active, the release of active cytokines such as IL-1β and IL-8 takes place. These cytokines are correlated with severe coronary artery disease. Furthermore, the NLRP3 inflammasome is upregulated after myocardial infarction [34,35]. Therefore, finding new molecules with higher potency and lower toxicity to modulate the inflammasome is a very important goal nowadays, because these types of illnesses are closely linked to unhealthy lifestyles [36]. Table 1 shows a list of nutraceutical compounds targeting inflammasomes in cardiovascular diseases.

Apigenin is a dietary flavonoid (4´,5,7, trihydroxyflavone) abundantly present in common fruits and vegetables, for instance oranges, grapefruits, parsley, onions, chamomile, wheat sprouts and some seasonings. It has anti-inflammatory activities as well as chemo-preventive ones. It has been reported that it protects endothelial cells from LPS-induced inflammation, but the mechanisms underlying its functions were unknown. Scientists have elucidated that apigenin inhibited inflammatory response by several mechanisms that all work together. Apigenin partially inhibits NLRP3 inflammasome by interrupting the Syk/Pyk2 pathway, as well as inhibiting NLRP3 and AIM2 inflammasome activation, but not NLRC4 [36]. Apigenin is implicated in the modulation of the inflammasome assembly by reducing the stability of the mRNA of pro-inflammatory cytokines and inhibiting ERK1/2 and NF_Ϗ_β activation in macrophages. In reference to the NLRP3 inflammasome, apigenin inhibits the oligomerization of ASC and interferes with its assembly in the cytoplasm, so that it cannot be fully activated. As a result, caspase-1 is not activated and no pro-inflammatory cytokines are released. Undoubtedly, targeting NLRP3 inflammasome represents an important therapeutic goal in terms of inflammatory diseases [36].

Parthenolide is a plant sesquiterpene lactone derived from a medicinal plant (feverfew), which is a small natural molecule that has been used as a herbal remedy for the treatment of many inflammatory diseases, for example arthritis, psoriasis and atherosclerosis [37,38,39]. Parthenolide is an inhibitor of the NF_Ϗ_β pathway that is initiated by the activation of TLRs, which is necessary for the induction of NLRP3 protein but not for its activation. However, its inhibitory properties are independent of the effects of NF_Ϗ_β activation. It is reported to be a NLRP3 pathway inhibitor at multiple levels; it inhibits the activation of caspase-1 by suppressing its catalytic activity through the direct alkylation critical cysteines of the p20 subunit and thus the activation of pro-inflammatory cytokines such as IL-1β cannot happen. The oligomerization and recruitment of ASC is crucial for NLRP3 activation and these are carried out by the NLRP3 ATP-asa activity in its NATCH domain. Partenolide inhibits the inflammasome by the inhibition of the ATP-asa activity and blocks the ASC recruitments. Therefore, the NLRP3 oligomerization, activation and the subsequent downstream events cannot occur. Parthenolide inhibits multiple components of the inflammasome pathways and previous studies revealed that it is a potent inhibitor of multiple inflammasomes, including NALP1 and NLRC4. A study where cells are exposed to the ligands that activate these inflammasomes (anthrax and salmonella, respectively) shows the capacity of parthenolide on this inhibition, not just the NLRP3 inflammasome [40].

Iridoids are secondary metabolites present in various plants, especially in species belonging to the *Apocynaceae, Lamiaceae, Loganiaceae, Rubiaceae, Scrophulariaceae* and *Verbenaceae* families. They involve a large family of bicyclic monoterpenes that have a large spectrum of pharmacological properties; they are anti-inflammatory, cardio-protective, anticancer, neuroprotective, hypoglycaemic and some have antioxidant properties [32,62]. Iridoids’ chemical structure consists of a six-membered ring with oxygen bound to a cyclopentane ring. They are classified in four groups: iridoid glycosides, non-glycosylated, secoiridoids (mostly glycosides) and bisiridoids. Scropolosise B and catalpol are iridoid glycosides isolated from the plants *Scrophularia dentata* Royle ex Benth. and *Rehmannia glutinosa* (Gaertn.) Libosch. ex Fisch. & C.A. Mey, respectively. In contrast to catalpol, Scropoloside B blocks the increase in IL-1β and TNFα levels and this is possible thanks to its capacity to suppress NF_Ϗ_β. Previous studies found that both inhibit the expression of NLRP3 mRNA and protein, due to their catalpol structure. It has been reported that Scropoloside B has a higher anti-inflammatory activity than catalpol, because of Scropoloside B’s greater capacity to block the increase in TNF-α and IL-1β in THP1 cells after being induced by LPS, with a 3-fold-increase. This may be explained by the fact that it contains the structure of catalpol and two phenylpropanoids. Due to its blocking of IL-1β, it could be an important treatment for diseases with high grades of inflammation, such as ischemic injury, atherosclerosis and type 2 diabetes [32]. On the other hand, genipin, an aglycone derived from iridoid glycoside geniposide and the main component of *Gardenia jasminoides* J.Ellis fruits, has been reported to have anti-inflammatory properties used to treat hypertension, diabetes and icteric hepatitis. It has been demonstrated that genipin suppresses the activation of NLRP3 and NLRC4 inflammasomes in vitro and in vivo, with the subsequent reduction in IL-1β, caspase-1 and ASC protein levels [43,62].

Gypenoside is a triterpenoid saponin (the mayor component of the *Gynostemma pentaphylla* (Thunb.) Makino), which has been reported to have anti-hypertensive, anti-hyperlipidemia, anti-hyperglycaemia, anti-aging and anti-inflammatory properties. In a high glucose stimulation model, which induces cardiomyocyte damage, researchers found a NLRP3 inflammasome ROS-dependent activation and its subsequent inhibition by the use of gypenoside. The use of this saponin has been proposed in order to treat myocardial damage due to its ability to inhibit NLRP3 inflammasome, and thus to release Il-1β and Il-18 in cardiomyocytes and in a mice model suffering from diabetes cardiomyopathy [44].

One of the most common pathogens causing heart failure is coxsackievirus B3, which is associated with acute myocarditis progression [63,64]. Researchers have reported high levels of inflammatory response as the main mechanism for cardiac injury [65]. Another iridoid glycoside, named morroniside, is one of the components of *Cornus officinalis* Siebold & Zucc. and was tested in myocardial injured-rats. Morroniside inhibited the NLRP3 inflammasome and downregulated the gene of ASC, caspase-3, IL-1 β and IL-18 [45]. Cornel iridoid glycoside, obtained from *Cornus officinalis* Siebold & Zucc., has beneficial actions in atherosclerosis, which may be a predisposing factor for suffering from an ischemic stroke. Its ability to inhibit IL-1, IL-6, TNF-α and prostaglandin E2 (in plasma) and NF_Ϗ_β [66,67] has been reported.

Korean red ginseng is one of the most commonly studied herbal medicines used around the world due to all its health properties. It is isolated from the roots of *Panax ginseng* C.A.Mey. and contains a large range of active components including saponins like ginsenoids and non-saponins like polysaccharides, peptides, fatty acids and mineral oils. The main active component of Korean red ginseng water extracts (RGE) is ginsenoids and these are widely used because of their anti-inflammatory properties, making them a remedy for many diseases such as cancer and cardiovascular and metabolic diseases [41]. It has been reported that ginsenoids from RGE are potent inhibitors of NLRP3 and AIM2 inflammasomes, but the key inhibitors are Rh1 and Rg3 ginsenoids. NLRP3 activation could be assessed by an elevation of ROS and intracellular Ca^+2^ and ginsenoids reverse this elevation, thus inhibiting NLR3 activation. Ginsenoids have an inhibitory effect on the ASC pyroptosome formation, meaning they impair the activation of AIM2’s ASC by dsDNA. Rg3 and Rh1 have anti-inflammatory properties. It has been reported that Rg3 alleviates obesity and myocardial injury in rodents and, moreover, Rh1 has been studied as an anti-carcinogenic agent [41].

Omega-3 fatty acids (also known as n-3 or ω-3) are polyunsaturated fatty acids (PUFA) that have been demonstrated to have many health benefits. Primarily, ecoisapentaenoic acid (EPA) and docosahexaenoic acid (DHA) can suppress inflammation and have a role to play in oxidative stress and have the ability to improve cellular function by changing gene expression. Consequently, they have beneficial roles in many inflammatory human diseases such as diabetes and arteriosclerosis [33,68]. It has been documented that the intake of these omega-3 fatty acids may decrease the expression of genes involved in inflammatory pathways such as NF_Ϗ_β. Both are associated with a reduced risk of suffering recurrent coronary artery events and sudden cardiac death after an acute myocardial infarction and they are linked with low levels of heart failure events. In addition, omega-3 fatty acids reportedly play a role in atherosclerosis and peripheral arterial disease (PAD). Omega-3 fatty acids can specifically inhibit NLRP3 and NLRP1b inflammasomes and the activation of caspase-1, and therefore inhibit the release of IL-1β [33].

Vascular system diseases are considered to be age-related because of their onset and progression. The process of aging is associated with endothelial dysfunction, impaired angiogenesis, defective vascular repair, arterial stiffening and remodeling, as well as atherosclerosis. All of them are contributors to the development of cardiovascular diseases. Endothelial senescence, which is an important contributor to age-related diseases, is linked to vascular pathologies and it is known to be implicated in the development and progression of CVDs. Cardiac endothelial cells, such as vascular smooth muscle, leukocytes and stem cells, as well as embryonic and hematopoietic cells, play an important role in the maintenance and regeneration of cardiovascular tissue. Therefore, damage to any of these cells may increase the probability of one suffering a cardiovascular disease [69]. The vast majority of human cells replicate themselves continuously and mitotically and when this is paused due to a stress and damage response, these cells enter a state called senescence. The cells suffer many changes in gene expression, morphology and functions, which may cause the progression of atherosclerosis. This process results in a phenotype that is pro-inflammatory, pro-thrombotic and pro-arteriosclerotic, leading to the loss of endothelial integrity, the vasodilatation capacity that leads to plaque formation, thrombosis and atherogenesis.

In this situation, the importance of finding effective methods to suppress endothelial senescence is necessary. A study performed by Sun et al. [69] demonstrated that natural anthocyanins-a food colorant derived from purple sweet potato (PSPC)-inhibited endothelial senescence via NLRP3 inhibition. These compounds have protective, anti-senescence and anti-inflammatory roles. They induce senescence by means of a high glucose treatment, which increases ROS levels and accelerates the accumulation of oxidative stress, which are ligands of NLRP3. Furthermore, the expression of caspase-1 (p. 10) is upregulated and pro-caspase-1 is under-regulated. All these findings suggest that the NLRP3 inflammasome is activated during senescence. However, these flavonoids inhibit NLRP3 and also the downstream reactions, such as the expression of ASC and caspase-1. These findings demonstrate that cyanidin acyl glucosides and peonidinacyl glucosides inhibit senesce by suppressing ROS levels and deactivating the NLRP3 inflammasome [34,35].

Quercetin is one of the most widely known and studied plant flavonoids, fundamentally for its powerful antioxidant capacity and beneficial effects on health. Quercetin is found in many fruits, vegetables, leaves, and grains. The biological effects of quercetin have been widely reported, including its anti-inflammatory, antioxidant and antitumor capacity [70]. The capacity of quercetin to regulate the NLRP3 inflammasome has also been reported, given its capacity to ameliorate kidney inflammation by blocking NLRP3 inflammasome activation and impairing caspase-1 and IL-1β expression [71]. Quercetin also inhibits both the NLRP3 and AIM2 inflammasomes by preventing ASC oligomerization and prevents interleukin-1-mediated mouse vasculitis [42].

Atherosclerosis, a leading cause of vascular disease worldwide, is a chronic and immunoinflammatory disease. A large portion of the population suffers from it, hence it is becoming the leading cause of mortality and morbidity in developed countries [72]. Inflammasome response occurs in the development of atherosclerosis and plays a decisive role in its complications. Curcumin, a natural polyphenolic compound, is the main active ingredient obtained from the roots of *Curcuma longa* L. and it is known for its anti-inflammatory, anti-infection and antioxidant properties [73]. Monocytes are known to be the main factor in the development of this disease, due to their invasion capacity of the atherosclerotic lesion and their following transformation into macrophages. In an in vitro study using PMA-induced macrophages, scientists have demonstrated the inhibitory effect of curcumin against NLRP3 inflammasome, suggesting that curcumin ameliorates the development and progression of atherosclerosis. It has been found that the double downregulation that curcumin exerts on TLR4/MyD88/NF_Ϗ_β signaling and on P2X7R expression leads to the inhibition of the NLRP3 expression and Il-1β and caspase-1 secretion [50].

*Penthorum chinense* Pursh is a traditional Chinese medicine herb whose main components are flavonoids, polyphenols, triterpenoids, lignans and organic acids [74,75]. Since inflammation is associated with atherosclerosis progression and the secretion of IL-1β is a critical factor for its pathogenesis [76,77], the use of the polyphenol thonningianin A (TA) extracted form *P. chinense* was tested in a mouse model of cardiovascular disease, showing a decrease in the NLRP3 and IL-1β expression [51].

Meanwhile, salvianolato, the main water-soluble bioactive compound found in the traditional Chinese herb *Salvia miltiorrhiza* Bunge, reportedly has anti-inflammatory and anti-fibrosis properties, which are linked to NLRP3 inhibition [78]. Nowadays, it has been used as a treatment for coronary heart disease in China [79]. Atrial fibrillation (AF) represents the most common cardiac arrhythmia suffered by millions of people worldwide and its incidence increases in those who also suffer from type 2 diabetes, arterial hypertension, coronary heart disease and heart failure [80]. Since AF is associated with inflammation and fibrosis, the anti-inflammatory and ant-fibrotic effects of salvianolato were tested in myocardial infarction rat models. In vivo studies showed high levels of NLRP3, pro-caspase and caspase-1, IL-1β, IL-18 and TXNIP expression; however, after the salvianolato treatment, there was a reduction in the expression of the aforementioned proteins. Recent studies have shown high levels of pro-inflammatory cytokines and NLRP3 inflammasome in patients suffering from AF, which can be used as a therapeutic approach for improving heart function [52]. On the other hand, tanshinone IIA is one of the main active lipophilic components found in *Salvia miltiorrhiza* Bunge and also has anti-atherosclerotic activity. It suppress NLRP3 inflammasome by inhibiting ROS and the lysosomal enzyme cathepsin B [55,81]. Researchers have determined that a derivate of it, named sodium tanshinone IIA, has anti-inflammatory, anti-antioxidant and cardio-protective effects. It plays a protective role in ischemia-induced myocardial injury by inhibiting the NLRP3 inflammasome through suppressing ROS and TXNIP [55].

*Oenanthe javanica* (Blume) DC. commonly known as water dropwort, is a plant that has been used in traditional Chinese medicine to treat hypertension, abdominal pain, fever, mumps and jaundice [82], and it has been used as an anti-arrhythmic [83] and anti-diabetic drug [84]. Isorhamnetin and hyperoside are two active compounds from *O. javanica* that have been studied as inflammasome inhibitors. Isorhamnetin was found to decrease the secretion of Il-1β, Il-18 and caspase-1 resulting from the activation of NLRP3 and AIM-2 inflammasomes. It also attenuates the expression of NLRP3 and pro-inflammatory genes, whereas hyperoxide also attenuates their secretion resulting from the activation of NLRC4 and AIM-2 inflammasome, but without altering gene expression [46]. Both nutraceuticals have been studied to be used as a pharmacological strategy to treat vascular diseases due to their anticoagulant and profibrinolytic properties [85].

Regarding coronary diseases, the moderate consumption of red wine has been attributed with lower incidences [86,87]. Due to the high levels of polyphenols contained in red wine, especially the antioxidant resveratrol, its use in moderation exerts cardiovascular benefits. Resveratrol has been reported to possess antihypertensive, anti-ischemia, anti-atherosclerotic and anti-heart failure activities, bestowing it with a powerful protective effect on cardiovascular health [49,88]. An in vitro study using resveratrol showed a decrease in IL-1β secretion and ASC and NLRP3 protein expression levels. However, better anti-inflammatory results were observed with the total red wine extracts due to a mixture of polyphenols, which enhance the biological effects more than resveratrol alone. Within these compounds, one can find: phenolic acids, such as gallic acid; stilbenes, such as resveratrol; catechins, such as catechin and epicatechin; and flavanols, such as quercetin [47].

Ilexgenin A is the main triterpenoid found in the leaves of *Ilex hainanensis* Merr. and has been reported to prevent lipid disorders [89]. As an endothelial dysfunction associated with lipids disorders, its beneficial effects in the vascular function have been studied. It was evidenced that ER stress disturbs endothelial homeostasis through TXNIP/NLRP3 induction; ER stress was induced in endothelial cells and then treated with ilexgenin A. It reduced the ROS levels with the subsequent reduction in the TXNIP induction and thus, NLRP3 inhibition. It also reduced the levels of the pro-inflammatory cytokines of Il-1β and IL-6, making the use of ilexgenin A a fantastic approach for ameliorating vascular dysfunction in ER stress conditions [53].

Many other nutraceuticals have been found to possess therapeutic potential in the treatment of cardiovascular diseases by attenuation of the NLRP3 inflammasome, such as luteolin [57] and dihydromyricetin [56] in atherosclerosis; and colchicine [58], triptolide [59], total flavones [60], umbelliferone [61] in ischemic myocardial disease.

## 6. Nutraceutical Compounds, the NLRP3 Inflammasome and Type 2 Diabetes

Type 2 diabetes, a chronic metabolic disorder, is the leading cause of mortality and morbidity worldwide and its cases are increasing constantly. One of the hallmarks of this disorder is a high inflammation rate and it has been shown that the NLRP3 inflammasome is deeply implicated in the development of insulin resistance in type 2 diabetes. On the other hand, diabetic nephropathy (DN) is a serious complication of diabetes mellitus, where persistent inflammation in circulatory and renal tissues is considered an important pathophysiological basis for the disease. NLRP3 and other inflammasome components are able to detect endogenous danger signals, resulting in the activation of caspase-1 as well as IL-1β, IL-18 and other cytokines, stimulating the inflammatory cascade reaction, which is crucial for DN [90].

Inflammation is the main cause of the development of many diseases, as explained above, and in terms of diabetes it is the main cause of the progression of obesity and insulin resistance. Impaired insulin signaling is the main problem that must be assessed in diabetic patients. It has been discovered that high levels of glucose cause oxidative stress, inflammation and mitochondrial dysfunction, where endoplasmic reticulum (ER) stress may contribute to all these factors, whilst excess fructose consumption causes a high prevalence of metabolic syndrome and inflammatory liver diseases [91]. In a research project, adipocytes and adipose tissue were exposed to high glucose levels in order to increase oxidative and ER stress. The latter initiates an inflammatory cascade where there is an increase in TXNIP expression, and therefore, NLRP3 is activated. NLRP3 activation involves the proteolytic cleavage of pro-caspase-1 and its activation and the production of the pro-inflammatory cytokine IL-1β. High levels of inflammation impair insulin IRS-1/PI3K/AKT/GLUT4 signaling. When adipose tissue is exposed to high glucose levels, IRS-1 is phosphorylated and AKT is dephosphorylated (both of them are inhibited) and thus there is a blockage in the insulin signaling, which leads to insulin resistance. In order to block this excessive inflammation event, the use of the two ginsenoids (outlined below) are used [92].

Ginseng is extracted from the root of *Panax ginseng* C.A.Mey. and has been used as a medicinal herb since ancient times in China, Korea and Japan. *Panax* means “all healing” since it was used as a natural remedy that helps to cure every disorder. Ginsenosides, which are triterpene saponins, are the most abundant component in ginseng and they impart its main pharmacological properties. Rb1 and its sub product, ginsenoside compound K, were tested by Chen et al. [92] in order to decrease the oxidative stress produced by high levels of glucose and inhibit the NLRP3 inflammasome, and thus, increase the sensitivity of insulin in adipose tissue (Table 2). The results showed that both ginsenoids attenuated the TXNIP expression and subsequent activation of NLRP3. In that way, the level of IL-1β was decreased and there was an increase in the sensitivity to insulin, as demonstrated by the reduction in IRS-1 phosphorylation, PI3K and AKT activation and then the insulin signaling was restored [92].

Furthermore, resveratrol was able to inhibit the activation of the TXNIP/NLRP3 axis both in adipocyte treated with high levels of glucose and in the adipose tissue of streptozotocin-induced diabetic mice through the suppression of ER stress and the inhibition of ROS-associated mitochondrial fission in an AMPK-dependent way, thus preventing high glucose-induced damage [97]. Similar results were found also for vitamin D3, whereby human retinal microvascular endothelial cells treated with high doses of glucose and in streptozotocin-induced Sprague-Dawley rats inhibited TXNIP/NLRP3 inflammasome pathway activation, thus exerting protective effects against retinal vascular damage and inflammatory-related pathologies, including diabetic retinopathy [98]. Finally, Xu et al. found that mangiferin, a glucosylxanthone that naturally occurs in mango, inhibited the activation of the TXNIP/NLRP3 inflammasome axis pathway, decreasing the expression of cleaved caspase-1 and the secretion of IL-1β secretion through the AMPK-induced suppression of ER stress in perivascular adipose tissue isolated from male Sprague-Dawley rats and from mice fed a high fat diet [99].

Omega-3 fatty acids (ω-3 FAs) have been studied as a nutraceutical remedy to treat inflammation disorders such a type 2 diabetes. The three most important ω-3FAs are α-linoleic acids (ALA), eicosapentanoic acid (EPA) and docosahexanoic acid (DHA), all of which are found in food, whether of vegetal or animal origin. A study conducted by Yiqing Yan et al. showed that ω-3FAs reduce the levels of pro-inflammatory cytokines such as IL-1β and are specific inhibitors of NLRP3 and NLRP1b inflammasomes [33]. By feeding mice a high fat diet, they reported that using DHA meant levels of glucose in plasma decrease, thus it may help to decrease insulin resistance in those mice. Doing the same experiment in NLRP3 KO mice, the beneficial effects of DHA were all abrogated. It is suggested that the way in which DHA brings this about is through the inhibition of NLRP3 [33].

Tocotrienols are natural compounds found in several vegetable oils, barley, wheat germ and some nuts and grains. They are members of the vitamin E family and, like tocopherols, they have antioxidant and anti-cancer activities. However, tocotrienols are unsaturated and possess an isoprenoid side chain, which allows them to penetrate into tissues. There are four types, of which γ-tocotrienol has high antioxidant and anti-inflammatory properties. A study conducted by Ahsan et al. demonstrated the NLRP3 inhibitory activity of tocotrienol in type 2 diabetes mouse models by two approaches: it impairs the NLRP3 priming by inhibiting NF_Ϗ_β and blocks NLRP3 activation. In this context, salvianolic acid A from *Salvia miltiorrhiza* Bunge was also able to alleviate atherosclerosis and type 2 diabetes in male Zucker diabetic rats on high fat diets, by decreasing hemoglobin A1C and C-reactive protein levels, improving lipid profile and aortic tissue condition, through the inhibition of NLRP3 inflammasome and NF-_Ϗ_B signaling activation [100].

The amounts of IL-1β and IL-18 are highly implicated in the development of insulin resistance, type 2 diabetes mellitus (T2DM) and obesity-associated inflammation. Harbis et al. [106] used APOE2 KI mice that lacked the protein responsible for the clearance of the high triglyceride content in LDL particles. These animals were fed a high fat diet (HFD), which leads to an increase in pro-inflammatory cytokines, such as IL-1β and IL-18, both of which cause high levels of systemic inflammation and insulin resistance. It has been demonstrated that high levels of plasma triglycerides promote systemic inflammation, which leads to an increase in the risk of suffering from metabolic disorders.

Abderrazak et al. found that arglabin (a natural sesquiterpene lactone mainly found in its isolated form *Artemisia glabella* Kar. & Kir.), was an important and promising natural molecule that normalizes plasma glucose and insulin by inhibiting the NLRP3 inflammasome. They also used APOE2 KI mice on a HFD and showed high levels of inflammation, which leads to the development of T2DM. Subsequently, these mice were treated with arglabin, which evidenced the inhibition of NLRP3 and its downstream cytokines, and thus the plasma levels of inflammatory cytokines decreased as well as the insulin resistance of the tissues, while T2DM improved. In addition, arglabin helps to protect β-pancreatic cells from apoptosis through the inhibition of NLRP3. All of these findings were demonstrated by generating APOE2/NLRP3 KI mice which, similar to arglabin treatment, showed a reduction in plasma glucose and insulin resistance in mice on a HFD [96]. Furthermore, it has been demonstrated that arglabin induces autophagy and it is thought that one of the ways in which NLRP3 is inhibited is by degrading it along with all of its components, such as pro-IL-1β, pro-caspase-1 and ASC. To conclude, NLRP3 can be seen as an important molecular target for treating T2DM [96]. In the same way, Lalitha et al. found that myricetin, the main flavanol in Horsegram (*Macrotyloma uniflorum* (Lam.) Verdc.) seed coat, decreased in the liver of streptozotocin-induced diabetic male Wistar rats, the gene expression of NLRP3, ASC and caspase-1, leading to a reduction in blood glucose levels and in liver dipeptidyl peptidase-4 and antioxidant enzyme activities, as well as leading to an improvement in plasma insulin levels [101]. Furthermore, other plant phenolic compounds exert similar effects: freeze-dried red raspberry powder enriched in polyphenols ameliorated levels of blood glucose, insulin resistance and glucose intolerance and the accumulation of hepatic lipids. It also suppressed the activation of NLRP3 inflammasome, caspase-1 and decreased the production of IL-1β and IL-18 in mice fed a high fat diet [102].

Aside from the aim of searching for nutraceuticals to ameliorate the consequences of type 2 diabetes, it is important to consider one of the main complications of diabetes: diabetic nephropathy (DN), which can lead to cardiovascular disease. Given that inflammation plays an important role in the development of DN, the need for finding natural compounds to target NLRP3 is a must. In a recent study, the anti-inflammatory properties of two alkaloids were used in order to decrease the protein level of NLRP3 and the huge quantities of pro-inflammatory cytokines in order to prevent DN progression. Piperine is an alkaloid phenolic compound, which is the main bioactive component of pepper, and Cepharanthine is a natural alkaloid extracted from the plant *Stephanie cepharantha* “*hayata”.* Abderrazak et al. [96] administered these two compounds in a rat model of diabetes and the results of the compounds and their combination showed a decrease in the mRNA and protein levels of TXNIP and NLRP3 in renal tissues. They also decreased levels of oxidative stress and NF_Ϗ_β activation. As explained above, TXNIP is a negative regulator of TRX, which is increased by high glucose levels and causes oxidative stress and inflammation by its binding to NF_Ϗ_β and NLRP3. Therefore, the strategy of decreasing its levels is an excellent approach towards stopping the progression of inflammatory diseases such as type 2 diabetes. Another study found that piperine reduces blood glucose levels as well as improving the sensitivity of insulin intake in type 2 diabetes [107]. In addition, given its capacity to increase the insulin-like growth factor-I (IGF-1), cepharanthine was shown to lower blood glucose and decrease insulin resistance [94]. Similar results were found for ginsenoside Rg5, whereby the kidneys of high-fat diet/streptozotocin-induced diabetic mice were able to decrease the expression levels of NLRP3, ASC and caspase-1, IL-1β and IL-18 as well as the expression of NF-kB and P38 MAPK phosphorylation, thus improving renal injury through the reduction in inflammation [103].

Scientists have proven that other nutraceuticals block DN, for instance curcumin. This is a highly pleiotropic molecule, which has antibacterial, anti-inflammatory, antioxidant, wound-healing, hypoglycemic and antimicrobial properties. It is isolated from the rhizome of *Curcuma longa* L. An experimental study discovered a high increase in the level of protein expression of NLRP3, caspase-1 and IL-1β in diabetic mice compared with their non-diabetic counterparts. After the administration of curcumin, the level of inflammation markers decreased. Additionally, this research was carried out in HEK-2 cells and the same result was observed. When HEK-2 cells were exposed to a high glucose concentration, levels of NLRP3, caspase-1 and IL-1β were greatly increased. However, when curcumin was added to the culture media, these levels were reduced. These results indicate the role of NLRP3 in the progression of DN and place NLRP3 inflammasome as a therapeutic target of the disease [95]. Curcumin, as well as allopurinol, also down-regulated TXNIP and inhibited NLRP3 inflammasome activation in fructose-fed rat livers and fructose-exposed BRL-3 A and HepG2 cells [91]. The same results were obtained when allopurinol was combined with quercetin in BRL-3A and human HepG2 exposed to high glucose, and in streptozotocin-induced diabetic rats. These nutraceutical compounds were able to inhibit the overexpression of TXNIP and the activation of NLRP3 inflammasome in order to decrease the levels of IL-1β and modulate the expression of proteins involved in lipid metabolism, contributing towards decreasing liver inflammation [105].

Lastly, another common complication of diabetes is foot ulcers, characterized by high levels of inflammation. Nutraceutical compounds can also be a valid tool in preventing this pathological condition thanks to their antioxidant and anti-inflammatory properties, as demonstrated by genistein, which ameliorated the fasting glucose levels, promoted wound closure, restored NLRP3, ASC and caspase-1 expression and improved the markers of inflammation such as NFkB, TNFα COX_2_ and iNOS, in alloxan-induced diabetic mice [104].

In conclusion, nutraceuticals represent a promising strategy for improving metabolic abnormalities, such as diabetes, thanks to their capacity to decrease inflammation by modulating NLRP3 inflammasome and its downstream pathways.

## 7. Nutraceutical Compounds, the NLRP3 Inflammasome and Neurological Diseases

Many people suffer from depression and cases of it are on the rise. It has been reported that neuroinflammation is involved in depression. Changfu Cao et al. [108] conducted a study in order to demonstrate these findings using a mouse model of chronic mild stress (CMS). As in depressive patients, the CMS mice showed a high level of pro-inflammatory cytokines, such as IL-1β and IL-18, in their hippocampi. In addition, the activation of NLRP3 is linked to clinical depression and can be ameliorated using antidepressant drugs [109]. Therefore, the need arose to test a nutraceutical compound as an antidepressant treatment (Table 3). The aforementioned research group measured levels of NLRP3 and ASC in mouse hippocampi and concluded that they were very high. However, when mice were treated with mangiferin, the levels of these molecules diminished as well as levels of pro-inflammatory cytokines. At that time, they tested the mice’s depression in a variety of ways, and it was highly reduced [108].

Apigenin, like mangiferin, can exert anti-inflammatory and neuroprotective actions upon depressive disorders. In a rat model of chronic unpredictable mild stress (CUMS), high levels of NLRP3 inflammasomes and caspase-1 were found, however when apigenin was used, these levels decreased. This inhibitory mechanism of the inflammatory process is partly due to the upregulation of PPARγ. In the promoter region of the NLRP3 and IL-1β genes, there is a PPARγ-binding-site; apigenin increases the expression level of PPARγ and thus it impairs the transcription of those inflammatory genes [110].

Sepsis-associated encephalopathy (SAE) is a pathology characterized by organ failure, brain cell damage and disturbances in neurotransmission. It is associated with severe brain dysfunctions and sepsis due to a high inflammatory process. It has been reported that in the hippocampi of septic mice, levels of IL-1β and NLRP3 are highly expressed, however when resveratrol is used, these levels decrease. Sirtuin 1 (Sirt1) is known for being a protein that it is implicated in the clearance of ROS and participates in many biological processes, such as aging, oxidative stress and inflammation. Resveratrol may develop its role through the enhancement of Sirt1 expression. Sirt1 inhibits the nuclear translocation of the p65 subunit to the nucleus by the deacetylation of NF_Ϗ_β, and thus resveratrol inhibits the NLRP3 priming and its subsequent activation [111].

Ischemic stroke is responsible for a high incidence of brain injury, in which inflammation is one of the mechanisms that leads brain cells to apoptosis and necrosis. Using a rat model of ischemic-reperfusion, high levels of IL-1β and IL-18 as well as NLRP3 expression were reported. In an ischemic stroke, oxidative stress is the main initiator and it has been shown that NLRP3 activation is achieved by means of a thioredoxin-interacting protein (TXNIP) (endogenous inhibitor of TRX), which is activated by oxidative stress. By administrating a plant-based natural antioxidant called Umbelliferone (UMB), which is a cumarin derivate, the levels of IL-1β, IL-18 and NLRP3 decrease. UMB decreases NLRP3 expression by two mechanisms—the reduction in TXNIP expression and the increase in PPAR levels [112].

In recent years, cerebral infarction has become one of the main causes of death. It has recently been discovered that a high level of inflammation causes pathogenesis; therefore, targeting the inflammation process could be an important approach in decreasing the severity of brain damage. Sulforaphane, which belongs to the isocyanate group of organosulfur compounds (4-methylsulfonylbutyl isothiocyanate) and is found in cruciferous vegetables, such as Brussels sprouts, broccoli and cabbages, was used in order to decrease the brain’s inflammation level after a cerebral stroke. It is a neuroprotective and anti-inflammatory compound, whose properties are exerted by its ability to inhibit the NLRP3 inflammasome and thus the production of pro-inflammatory cytokines, such as IL-1β and IL-18 [113].

Cognitive impairments have been linked to cerebrovascular pathologies and cerebral ischemia is regarded as the main manifestation of circulatory diseases, since an ischemic stroke increases the risk of suffering a coronary heart disease and vice versa. NLRP3 inflammasome plays a crucial role in ischemic stroke and it has been found that it is increased in ischemic conditions [114]. Astragaloside IV has been reported to attenuate NLRP3 and caspase-1 activation and decrease IL-1β and TNF-α release in a mouse model of ischemia and reperfusion. These results show the ability of astragaloside IV to improve neurological impairments after transient cerebral ischemia and reperfusion by its anti-inflammatory properties [115]. Ruscogenin, a steroidal sapognin obtained from *Ophiopogon japonicus* (Thunb.) Ker Gawl., has been shown to have protective effects after ischemic stroke by inhibiting NLRP3, IL-1β, caspase-1 and TXNIP in vitro and in vivo [116]. Sinomenine is an alkaloid with anti-inflammatory properties, which has been shown to bring about improvements in treatment for ischemic stroke in an experimental cerebral ischemia mouse model and OGD-treated cell model. It inhibits the activation of NLRP3, ASC and caspase-1 via the AMPK cellular pathway [117]. Another nutraceutical that has been reported to protect against cerebral ischemia reperfusion in rats by inhibiting NLRP3 is arctigenin. Scientists have found a reduction in infarct volume, a decrease in brain water content and an improvement in neurological scores due to the inhibition of NLRP3 activation, which is SIRT1-dependent, and Il-1β and IL-18 release in vitro and in vivo [118]. It has been reported that resveratrol ameliorates cerebral I/R injury and shows neuroprotective effects by inhibiting NLRP3 inflammasome activation through Sirt1-dependent autophagy activity. Resveratrol is an inducer of SIRT1 expression, and it carries out its function through a mechanism dependent on the activation of SIRT1. Resveratrol increases the expression of SIRT1, which continues with an increase in the autophagy pathway and then NLRP3 expression is suppressed. In addition, there was a decrease in the activation of caspase-1, Il-β and IL-18 [119].

As mentioned above, one of the most common brain injuries worldwide is brain ischemia, which can lead to neuronal damage and cell death. The brain is an organ, which needs high supplementation, so an ischemia event causes neuronal death because of the high extrasynaptic glutamate release, causing brain neurotoxicity. It is thought that this release is responsible for the induction of ER stress, leading to inflammation. In response to ER stress, TRX allows TXNIP to interact with NLRP3, activating an inflammatory cascade where the maturation and secretion of IL-1β takes place. In brain tissues, it leads to neuronal cell death. Curcumin is widely known for its anti-inflammatory and neuroprotective properties. In a research project conducted on mice, their hippocampi were stimulated with glutamate in order to discover how curcumin acts to protect the brain [120]. To recreate ischemic injury, mice hippocampi were deprived of oxygen and glucose and after that, high levels of glutamate and IL-1β were found. However, when they were treated with curcumin, there was a significant reduction in IL-1β and glutamate. Expressions of TXNIP and NLRP3 were also measured and a reduction was noted following the treatment. Using cells transfected with AMPKα1/2-specific siRNA, they found that the inhibitory action of curcumin on TXNIP and NLRP3 is dependent on the AMPK action. In summary, brain ischemia leads to the release of extrasynaptic glutamate that induces ER stress and TXNIP/NLRP3 activation. Curcumin suppresses ER-stress and downregulates TXNIP/NLRP3 with the regulation of AMPK activity [120]. Similar results were found also for chrysophanol, a member of the anthraquinone family, extracted from plants of the Rheum genus, with well-known biological properties, such as anti-inflammatory activity. In male CD1 mice subjected to transient middle cerebral artery occlusion, it improved neurological deficits and tissue injury, ameliorating brain edema, infarct volume and decreased inflammasome, through the reduction in NALP3, ASC, caspase-1, and IL-1β expression [121].

It has been demonstrated that inflammasomes play an important role in the development of many neurodegenerative diseases such as Alzheimer’s disease (AD) and Parkinson’s disease (PD). In AD, there exists an elevated level of inflammation called neuroinflammation, which activates microglia and astrocytes and the recruitment of immune cells. It contributes to the disease’s progression and thus to neuronal loss [124]. It has been reported that one of the factors that increases the generation and development of AD is high levels of glucocorticoids (stress hormone). Many studies have shown that high levels of plasma cortisol help the development of AD and its plasma levels are highly linked to the progression of dementia. Glucocorticoids (GCs) are widely known because they impair learning and memory, alter neuronal plasticity, produce atrophy in several brain areas, reduce hippocampal dendritic complexity and promote hippocampal cell death. Zhang et al. [122] treated mice with 5 mg/kg dexamethasone (DEX) (a synthetic glucocorticoid) for 28 days to increase neuroinflammation and induce neurodegeneration through NLRP1 activation. Neuroinflammation is known as a cause of cognitive declaims during aging and in degenerative diseases such as AD. The use of Rg1, a ginsenoside that has neuroprotective effects, decreases the activation of NLRP1, in turn activated by chronic DEX exposure that causes hippocampal and neuronal damage. After 28 days of DEX exposure, they detected high expression levels of NLRP1, caspase-1 and 5, ASC, IL-1β and IL-18. However, when the mice were treated with Rg1 (2 and 4 mg/kg) there was a decrease in the expression of all of the aforementioned inflammatory markers. In addition, Rg1 increases the expression of the glucocorticoid receptors (GR) and thus, decreases the IL-1β and IL-18 levels. Using this natural molecule, an improvement in learning and memory was found, alleviating the cognitive dysfunction caused by GC. To conclude, Rg1 suppresses the neuroinflammation caused by GC exposure through the inhibition of NLRP1 and the increase in GR and thus improves cognitive function [122]. Recently, it has been shown that astaxanthin and especially its acylated form with docosahexaenoic acid may be a possible therapeutic tool for AD, since their administration to APP/PSEN1 double-transgenic mice reduced oxidative stress, neuroinflammation and the expression of inflammasome proteins and thus decreased the cognitive disorders [123].

## 8. Nutraceutical Compounds, the NLRP3 Inflammasome and Cancer

Chronic inflammation has long been associated with various types of cancer and closely related to different stages of cancer development. During malignant transformation or cancer therapy, it has been hypothesized that inflammasomes become activated in response to danger signals produced by the tumors, or from therapy-induced damage to the tumor or healthy tissue being identified as a positive regulator of tumor cell proliferation and metastasis [125]. Several researchers report the effects of nutraceuticals, mainly polyphenols, on cancer [126], as well as the capacity of some flavonoids to suppress the NLRP3 inflammasome [127] (Table 4); however, studies regarding the relationship between nutraceuticals, NLRP3 inflammasome and cancer are still scarce.

Epigallocatechin-3-gallate (EGCG), the main polyphenolic found in green tea, has been demonstrated to possess anti-inflammatory, antioxidant, anti-mutagenic and anti-carcinogenic properties. EGCG inhibits various inflammatory enzymes and cytokines (iNOS, COX_2_, MMPs, IL-6, IL-8, IL-12 and TNFα), which are induced by secreted active IL-1β [128,129]. It has been proposed that EGCG has an anti-tumor effect on human melanoma cells (HMC) due to its inhibitory effect on proliferation. EGCG suppresses NF-κB activity and reduces IL-1β secretion. The decreased IL-1β is associated with the downregulation of NLRP1, a component of the inflammasomes, and reduced caspase-1 activation [128]. The authors demonstrated that the inhibitory effect of EGCG on tumor proliferation was abolished by silencing NLRP1, suggesting the role of inflammasomes in the tumor-inhibitory effect of EGCG in HMC. However, the researchers did not demonstrate NLRP3 inhibition, in contrast with a previous study that demonstrated the preventive effects of EGCG in lupus nephritis mice via NLRP3 inhibition [130]. This discrepancy, according to the authors, could result from different cell types (renal cortex cells vs. melanoma cells) and the different doses of EGCG used. The mechanisms by which EGCG inhibits inflammasomes are not completely known. Previous studies have identified a 67-kDa laminin receptor as a cell surface receptor for EGCG [131], which could be related to inflammasome downregulation.

Luteoloside (luteolin-7-O-glucoside; cynaroside), can be found in dandelion root coffee (*Taraxacum officinale* (L.) Weber ex F.H.Wigg.) and in artichoke (*Cynara scolymus* L.) [140]. It has anti-inflammatory [141], free radical scavenging [142] and antibacterial properties [143]. Resaerchers have reported that luteoloside suppresses hepatocellular carcinoma cells (HCC) through the inhibition of HCC migration and invasion and has the capacity to downregulate the expression level of NLRP3, caspase-1 and IL-1β, suppressing proliferation and metastasis of HCC [132].

Curcumin is a natural polyphenol extracted from *Curcuma longa* L. rhizomes, well-known for its anti-tumor properties against a wide range of cancers, due to its relevant antioxidant, anti-inflammation and pro-apoptotic potential. In malignant mesothelioma cells, curcumin has been shown to downregulate the expression of genes related to inflammasomes, such as TLR, NF-κB and IL-1β, as well as to induce pyroptosis by activating caspases and promoting the release of High Mobility Group Box1 (HMGB1) [134].

Berberine is a natural alkaloid extracted from many traditional Chinese herbs, with several biological activities, including anti-microbial, anti-inflammatory and anti-diabetic potential, as well as cerebrovascular and cardiovascular protection. Recently, Yao et al. found that in triple-negative MDA-MB-231 breast cancer cells, berberine was able to inhibit the pathway of NLRP3 inflammasome, by reducing the mRNA expression of NLRP3, caspase-1 and IL-1β and decreasing the protein expression of P2X7, NLRP3, pro-caspase-1 and ASC in a dose-dependent manner [135].

Two other nutraceuticals isolated from Chinese herbs are promising candidates for counteracting cancer through the activation of the NLRP3 pathway. Indeed, in non-small-cell lung cancer, polyphyllin VI extracted from *Trillium tschonoskii* Maxim. [136] and Huaier extract (a kind of fungus from *Trametes robiniophila* Murr. [137]) were able to induce pyroptotic cell death through the activation of caspase -1 by increasing the levels of ROS, which in turn activated the NF-κB and the NLRP3 signal pathways, providing new insights into the relationship between nutraceuticals, inflammasomes and cancer. Similar results were recently reported for anthocyanin by Yue et al. [138] in oral squamous carcinoma cells via anthocyanin-activated pyroptosis, leading to cell death, by increasing the gene and the protein expression of NLRP3, caspase-1 and IL-1β and, more interestingly, these results were abolished with the use of caspase-1 inhibitors.

Finally, in myeloid-derived suppressor cells, the omega-3 fatty acid DHA enhanced the efficacy of 5-fluoruracile, one of the most commonly used drugs in treating solid cancer by inhibiting the assembly of the NLRP3 and JNK pathways through the activation of β-arrestin-2, with a consequent decrease in IL-1β secretion [139].

Isorhamnetin (3-methylquercetin) is an *O*-methylated flavanol from the class of flavonoids. Common food sources of this 3’-methoxylated derivative of quercetin and its glucoside conjugates are: pungent yellow or red onions; Mexican tarragon (*Tagetes lucida* Cav.), used as a spice, medicinal herb and hallucinogenic; and an efficient flavonoid compound isolated from sea buckthorn (*Hippophae rhamnoides* L.) [133]. In addition to its known functions as a powerful antioxidant, the capacity of isorhamnetin to inhibit inflammasome activation was recently reported [46]. In bone marrow-derived macrophages (BMDMs), isorhamnetin attenuated the secretion of IL-1β resulting from NLRP3, NLRC4, and AIM2 inflammasome activation without interrupting the cytokine transcription. Isorhamnetin selectively inhibited NLRP3 and AIM2 inflammasome activation and down-regulated the expression of pro-inflammatory cytokines.

Since chronic inflammatory responses are closely associated with various types of cancer and their stages of development, the action of the different nutraceuticals on inflammasomes could serve as a basis for studying the regulation of the different inflammasomes by these compounds and their possible involvement in regulating the effect of inflammasomes on cancer. Therefore, these results are promising when analyzing the possible relationship between the effect of nutraceuticals on NLRP3 and the capacity of NLRP3 as a regulator of tumor cell proliferation.

## 9. Conclusion and Perspectives

Recent studies have uncovered a novel function of inflammasomes in protecting the organism through the activation of anti-inflammatory defense pathways. Abundantly expressed, inflammasomes appear as a precursor to the inflammatory response through activating the production of several cytokines. However, the pathological consequences that inflammatory processes may have beyond their defense and repair action have been related to different common diseases associated with a high level of inflammation where inflammasomes are activated. In this context, nutraceuticals have emerged as an alternative against those inflammatory processes that an excess in inflammatory response could cause. The proven anti-inflammatory properties of many of these compounds are one of the main action mechanisms by which they exert their regulatory or inhibitory action on inflammasomes, as well as their potent role against oxidative stress damage and the ability to improve cellular function by changing gene expression

Since inflammation, and particularly the mechanism of activation of inflammasomes, is closely involved in aging and important human diseases, such as degenerative, cardiovascular, neurological and metabolic disorders, and cancer, the regulatory action of certain nutraceuticals on the inflammasomes complex might provide new possibilities for the treatment of such diseases. As always, new findings often lead to new questions. For example, what is the direct role of the most common bioactive dietary compounds, and what is the regulation of the inflammation related to cardio-vascular, degenerative, neurological, metabolic disorders and cancer? Can nutraceuticals be used to treat inflammation-related diseases? And, if so, what would be the best approach: protein complex, cell, or nutrition? Future studies into these areas may offer novel and useful insights. 

## Figures and Tables

**Table 1:** List of Nutraceutical Compounds targeting Inflammasomes in Cardiovascular Diseases.

Nutraceutical Compound	Classification/Source	Overall Role in Inflammasomes	Experimental Model	Molecular Mechanism	Ref.
Apigenin	Flavonoid/Citrus fruits, vegetables	NLRP3, AIM2 inhibitor	-Human THP1 cells -Mouse J774A.1 macrophage -HEK-293 cells	1. Syk/Pyk2 pathway interruption 2. Inhibits ERK1/2 and NF_Ϗ_β activation in macrophages 3. Inhibits oligomerization of ASC and interferes with its assembly in the cytoplasm 4. No activation of caspase 1	[30,36]
Parthenolide	Sesquiterpene lactone/*Tanacetum parthenium* (L.) Sch. Bip.	NLRP3, NALP1, NLRC4 inhibitor	-LPS-induced inflammation in NG5 cell line mouse bone marrow cells	1. Inhibits NF_Ϗ_β 2. Inhibits oligomerization and ASC recruitments 3. Inhibits NLRP3 and caspase 1	[40]
Scropoloside B	Iridoids glycosides/*Scrophularia dentata* Royle ex Benth.	NLRP3 inhibitor	-HEK293 cells -Human THP1 cells	1. Inhibits NF_Ϗ_β 2. Decreases the expression of NLRP3 and Il-1β	[32]
Catapol	Iridoids glycosides/*Rehmannia glutinosa* (Gaertn.) Libosch. ex Fisch. & C.A. Mey	NLRP3 inhibitor	-HEK293 cells -Human THP1 cells	1.Decreases the expression of NLRP3	[32]
Rh1 and Rg3	Ginsenoside/*Panax ginseng* C.A.Mey.	NLRP3, AIM2 inhibitor	-LPS-induced inflammation in bone marrow-derived macrophages (BMDMs) and THP-1 cells -LPS-induced inflammation in Male C57BL/6 mice (8-week-old)	1.Inhibits the NLRP3 and AIM2 expression 2.Inhibits ASC pyroptosome formation 3.Inhibits caspase 1 activation and the secretion of IL-1	[41]
DHA	ω-3FAs/Fish, Crustaceans, Molluscs, Eggs	NLRP3, NLRPb1 inhibitor	-Mouse model -Human THP1 cells	1. Decreases the expression of genes involved in the inflammatory pathways of NF_Ϗ_β 2. Inhibits the activation of caspase 1 and the release of IL-1β	[33]
PSPC	Flavonoid/Fruits, vegetables, leaves and grains	NLRP3 inhibitor	-Male ICR mice -HUVECs	1. Suppress ROS level 2. Downregulation of pro-caspase1	[34]
Quercetin	Flavonoids/Fruits, vegetables, leaves, and grains	NLRP3, AIM2 inhibitor	-Vasculitis model in C57BL/6 mice	1. Impaired expression of caspase-1 and IL-1β 2. Prevention of ASC oligomerization	[42]
Puerarin and Troxerutin	Isoflavone/Root of *Pueraria lobata* (Willd.) Ohwi Flavonoid*/Sophora japonica L.*	NLRP3 inhibitor	-HUVECs cells	1. Decreases NLPR3, Il-1B and casapase-1 levels	[34]
Genipin	Iridoids glycosides/*Gardenia jasminoides* J.Ellis	NLRP3, NLRC4 inhibitor	-Mouse model -BMDMs cells	1. Inhibits NLRP3 and NLRC4 inflammasomes 2. Decreases Il-1β, caspase-1 and ASC protein levels	[43]
Gypenoside	Triterpenoid saponin/*Gynostemma pentaphylla* (Thunb.) Makino	NLRP3 inhibitor	-H9C2 cells -SD rats	1. Inhibits NLRP3 inflammasome 2. Decreases Il-1β and IL-18 protein levels	[44]
Morroniside	Iridoid glycoside/*Cornus officinalis* Siebold & Zucc.	NLRP3 inhibitor	-SD rats	1. Inhibits NLRP3 2. Downregulation of ASC, caspase-3, Il-1β and IL-18	[45]
Isorhamnetin and Hyperoside	Flavonoids/Water dropwort *Oenanthe javanica* (Blume) DC.	NLPR3, AIM-2 inhibitor	-Bone marrow-derived macrophages (BMDMs) form C57BL/6 mice -THP1 cells	1.Decreases the of Il-1β, IL-18 and caspase-1 secretion	[46]
Resveratrol	Stilbene (flavonoid)/Skin of grapes, blueberries, raspberries, cmulberries and red wine	NLRP3 inhibitor	-J774A.1 cells -Raw 264.7 cells -Sprague–Dawley rat	1. Decreases the secretion of Il-1β 2. Decreases the ACS and NLRP3 proteins 1. Suppresses NF_Ϗ_β and inhibits NLRP3 1.Supresses IL-1β and IL-18 2.Decreases NLRP3 and caspase-1 expression	[47] [48] [49]
Curcumin	Polyphenol/*Curcuma longa* L. roots	NLRP3 inhibitor	-THP1 cells -PMA-induced macrophages	1. Decreases NLRP3 expression and Il-1β and caspase 1 secretion through the inhibition of TLR4/MyD88/ NF_Ϗ_β signalling and P2X7R expression	[50]
Thonningianin A	Polyphenol/*Penthorum chinense* Pursh	NLRP3 inhibitor	-ApoE-KO mice	1. Decreases NLRP3 and Il-1β expression	[51]
Salvianolato	Polyphenol/*Salvia miltiorrhiza* Bunge	NLRP3 inhibitor	-SPF Sprague-Dawley rats	1. Decreases NLRP3, pro-caspase1, caspase-1, Il-1β, IL-18 and TXNIP expression	[52]
Ilexgenin A	Triterpenoid/*Ilex hainanensis* Merr.	NLRP3 inhibitor	-EA.hy-926 cells -Primary rat vascular endothelial cells (VECs)	1. Decreases the TXNIP/NLRP3 activation under ER stress condition	[53]
Tanshinone IIA and sodium tanshinone IIA	Diterpenoid/*Salvia miltiorrhiza* Bunge	NLRP3 inhibitor	-RAW264.7 macrophages -Beagle dogs	1. Decreases IL-1β levels 1. Inhibits the generation of ROS and TXNIP 2. Decreases the NLRP3 activation and the secretion of IL1-β and IL-18	[54] [55]
Dihydromyricetin	Flavonoid/*Ampelopsis grossedentata* (Hand.-Mazz.) W.T.Wang	NLRP3 inhibitor	-HUVECs	1. Attenuates NLRP3 inflammasome	[56]
Luteolin	Flavonoid/*Reseda luteola* L.	NLRP3 inhibitor	-RAW264.7 cells	1. Inhibits NLRP3 inflammasome 2. Decreases TNF-α and IL-6 levels	[57]
Colchicine	Alkaloid/*Colchicum autumnale* L.	NLRP3 inhibitor	-ACS patients	1. Suppresses NLRP3 inflammasome 2. Decreases Il-1β, IL-6 and IL-18 levels	[58]
Triptolide	Diterpenoid/*Tripterygiumwilfordii* Hook F.	NLRP3 inhibitor	-C57/BL6 mice	1. Inhibits the NLRP3 inflammasome 2. Inhibits IL-1β, IL-18, MCP-1 and VCAM-1 release	[59]
Total flavones	Flavonoids/*Abelmoschus manihot* (L.) Medic	NLRP3 inhibitor	-I/R Rats	1. Inhibits NLRP3 inflammasome 2. Decreases the IL-1β, IL-6 and TNF-α levels	[60]
Umbelliferone	Phenolic coumarin/*Rutaceae* and *Umbelliferae*	NLRP3 inhibitor	-Sprague-Dawley rats	1. Inhibits the NLRP3 inflammasome and IL-6 and TNF-α levels	[61]

BMDMs: Bone marrow-derived macrophages; ASC patients: acute coronary syndromes patients; HUVECs: Human umbilical vein endothelial cells; PSPC: Purple sweet potato color; SD: Sprague-Dawley; NF_Ϗ_β Nuclear factor _Ϗ_β; ROS: reactive oxygen species.

**Table 2:** List of Nutraceutical Compounds targeting Inflammasomes in Type 2 Diabetes.

Nutraceutical Compound	Classification/Source	Overall Role in Inflammasomes	Experimental Model	Molecular Mechanism	Ref.
Ginsenoside Rb1 and Ginsenoside CK	Triterpene saponins/*Panax ginseng* C.A.Mey. root	NLRP3 inhibitor	-3T3-L1 adipocyte cells -Mouse model	1. Inhibits NLRP3 inflammasome 2. Attenuation of TXNIP expression 3. Reduction in IL-1β expression 4. Reduction in IRS-1 phosphorylation and PI3K and AKT activation	[92]
γ-Tocotrienol	Isomers unsaturated Vitamin E/Fruits, vegetables, nuts, meats, cooking oils and some grains	NLRP3 inhibitor	-Mouse model of type 2 diabetes	1. Inhibits NF_Ϗ_β 2. Inhibits NLRP3 activation	[93]
DHA	Omega 3 Fatty acids (ω-3FAs)/Animal and plant origin	NLRP3, NLRP1b inhibitor	-Human THP1 cells -Mouse model	1. Decreases the expression of genes involved in the NF_Ϗ_β inflammatory pathways 2. Inhibits caspase1 activation and thus inhibits IL-1β release	[33]
PiperineCepharanthine	Alkaloid/Black pepper/*Stephania cepharantha “*Hayata*”*	NLRP3 inhibitor	-Diabetic nephropathy model in adult male (SD rats)	1. Decreases the levels of oxidative stress and activation of NFKβ 2. Decreases levels of TXNIP and NLRP3 mRNA and proteins in kidney tissues 3. Increase insulin-like growth factor-I (IGF-1)	[94]
Curcumin	Flavonoid/*Curcuma longa* L.	NLRP3 inhibitor	-C57BL/KsJ db/db (diabetic) mice model HK-2 cells	1. Decreases the NLRP3 i, capase1 and IL-1B expression	[95]
Arglabin	Sesquiterpene lactone/*Artemisia glabella* Kar. & Kir.	NLRP3 inhibitor	-INS-1 cells -ApoE2Ki mice	1. Degrading NLRP3 and pro-IL-1β, pro-caspase 1 and ASC	[96]
Resveratrol	Stilbene/Skin of grapes, blueberries, raspberries and mulberries	NLRP3 inhibitor	-3T3-L1 adipocytes -Streptozotocin-induced diabetic mice (ICR male mice)	1. Decreases TXNIP levels and inhibits cleavage caspase-1 induction 2. Reduced release of IL-1β	[97]
Vitamin D3	Cholecalciferol/Fish, beef, cheese, egg yolk	NLRP3 inhibitor	-HRMECs -Streptozotocin-induced SD rats	1. Decreases the TXNIP levels 2. Decreases NLRP3 activation	[98]
Mangiferin	Naturally occurring glucosylxanthone/Mango	NLRP3 inhibitor	-Perivascular adipose tissue isolated from male SD rats and from high-fat diet feeding in mice	1. Decreased levels of TXNIP and inhibition of cleaved caspase-1induction 2. Reduced release of IL-1β	[99]
Salvianolic acid A	Propanoic acid/*Salvia miltiorrhiza* Bunge	NLRP3 inhibitor	-Male Zucker diabetic fatty rats	1. Inhibits NF_Ϗ_β 2. Inhibits NLRP3 activation	[100]
Myricetin	Flavonoid/Horsegram seed coat (*Macrotyloma uniflorum* (Lam.) Verdc.)	NLRP3 inhibitor	-Streptozotocin-induced diabetic male Wistar rats	1. Decreases the expression of NLRP3, ASC and Caspase-1	[101]
Polyphenols	Polyphenol/Freeze-dried red raspberry	NLRP3 inhibitor	-High-fat diet feeding C57BL/6 mice	1. Decreases NLRP3 and caspase-1 levels 2. Decreases IL-1β and IL-18 production	[102]
Ginsenoside Rg5	Ginsenoside/*Panax ginseng* C.A.Mey.	NLRP3 inhibitor	-High-fat diet/streptozotocin-induced diabetic mice (C57BL/6 mice)	1. Decreases the expression of NLRP3, ASC and Caspase-1 2. Decreases the expression of IL-1β and IL-18 3. Decreases of NF_Ϗ_β and P38 MAPK phosphorylation	[103]
Genistein	Isoflavone/Legumes	NLRP3 activator	-Alloxan-induced diabetic ICR mice	1. Restored expression levels of NLRP3, ASC and Caspase-1 2. Improves the levels of NF-_Ϗ_β, NF_Ϗ_β, TNFα COX_2_ and iNOS	[104]
Curcumin+Allopurinol	Flavonoid/*Curcuma longa* L.	NLRP3 inhibitor	-BRL-3A cells and -Human HepG2 cells exposed to high fructose -Fructose-fed rat (Male SD rats)	1. Decreases overexpression of TXNIP via up-regulating miR-200a	[91]
Quercetin+Allopurinol	Flavonoid/Found in many fruits, vegetables, leaves and grains	NLRP3 inhibitor	-BRL-3A and -Human HepG2 exposed to high glucose -Streptozotocin-induced diabetic rats (Male SD rats)	1. Decreases overexpression of TXNIP 2. Reduces expression of IL-1β 3. Modulates the expression of proteins involved in lipid metabolism	[105]

Glc: β-D-glucopyranosyl; DHA: docohexanoic acid; Compound K: Ginsenoside CK; HRMECs: Human retinal microvascular endothelial cells; SD: Sprague-Dawley; NF_Ϗ_β: Nuclear factor-_Ϗ_β. NF_Ϗ_β

**Table 3:** List of Nutraceutical Compounds targeting Inflammasomes in Neurodegenerative Diseases.

Nutraceutical Compound	Classification/Source	Overall Role in Inflammasomes	Experimental Model	Molecular Mechanism	Ref.
Magniferin	Poliphenol of C-glucosylxanthone/*Mangifera indica* L. (Mango tree)	NLRP3 inhibitor	-CMS in mice	1. Inhibits hippocampal NLRP3 inflammasome 2. Inhibits caspase-1/Il-1β axis 3. Decreases the ASC expression 4. Decreases the Il-18 production	[108]
Apigenin	Flavone/Citrus fruits, vegetables	NLRP3 inhibitor	-Rat model of chronic unpredictable mild stress (CUMS)	1. Increases the expression levels of PPARγ 2. Decreases the NLPR3, Il-1B and casapase-1 levels	[110]
Resveratrol	Stilbene/Skin of grapes, blueberries, raspberries and mulberries	NLRP3 inhibitor	-C57BL/6 mouse model -Mouse BV2 cells -MCAO-injury rats	1. Decreases the NLRP3 generation via activation of SIRT1 2. Downregulates the level of IL-1β and IL-18 3. Decreases the NF_Ϗ_β levels	[111]
Umbelliferone	7-hydroxycoumarin/Plants: *Rutaceae* and *Apiaceae* families Carrot, coriander, garden angelica	NLRP3 inhibitor	-Rat model of ischemic reperfusion (SD rats)	1. Decreases the TXNIP expression 2. Increases the PPARγ levels 3. Inhibits NLRP3 inflammasome	[112]
Sulphoraphane	Isothiocyanate/Broccoli, Brussels sprouts, cabbages	NLRP3 inhibitor	-Brain ischemia/reperfusion injury model in adult male (SD rats)	1. Suppresses I/R-induced NLRP3 inflammasome expression 2. Downregulation of cleaved caspase-1 3. Reducing IL-1β and IL-18 expression	[113]
Curcumin	Pigment from tumeric*/Curcuma longa* L.	NLRP3 inhibitor	-INS-1 cells -ApoE2Ki mice	1. Inhibits hippocampal NLRP3 inflammasome 2. Downregulates TXNIP/NLRP3 with the regulation of AMPK activity	[120]
Rg1	Ginsenoside/*Panax ginseng* C.A. Mey *Panax japonicus* (T.Nees) C.A. Mey.	NLRP1 inhibitor	-ICR mice	1. Reduces expression levels of NLRP1, caspase 1 and 5, ASC and IL-1β and IL-18 2. Increases expression of the glucocorticoid receptors	[122]
Astragaloside-IV	*Astragalus membranaceus* (Fisch.) Bunge	NLRP3 inhibitor	-ICR mice	1. Attenuates NLRP3 2. Decreases IL-1β and TNF-α levels 3. Decreases NF_Ϗ_β translation	[115]
Ruscogenin	Steroidal sapognin*/Ophiopogon japonicus* (Thunb.) Ker Gawl	NLRP3 inhibitor	-bEnd.3 cells-C57BL/6J mice	1. Inhibits NLRP3, IL-1β, caspase-1 and TXNIP expression	[116]
Sinomenine	Alkaloid*/Sinomenium acutum* (Thunb.) Rehder & E.H.Wilson	NLRP3 inhibitor	-MCAO mice model -OGD cell model (Primary mixed glial cells)	1. Inhibits the NLRP3 via AMPK pathway 2. Inhibits ASC and caspase-1	[117]
Arctigenin	*Lignan/Arctium lappa* L.	NLRP3 inhibitor	-MCAO-injury rats -OGD-injury EX527 cells	1. Decreases the NLRP3 generation via activation of SIRT1 2. Downregulates the level of IL-1β and IL-18	[118]
Asthaxantin	Carotenoid/marine organisms, such as crab, salmon, shrimp, krill and microalgae	NLRP3 inhibitor	-PSEN1(APP/PS1) double-transgenic mice	1. Decreases the ASC expression 2. Reduces the IL-1β and TNF-α levels	[123]
Chrysophano	Anthraquinone/Rheum genus	NLRP3 inhibitor	-MCAO Male CD1 mice	1. Decreases the NLRP3 and ASC expression 2. Reduces the IL-1β and caspase 1 expression	[121]

PPARγ: peroxisome proliferator-activated receptors γ; Sirt1: Sirtuin1; TXNIP: thioredoxin-interacting protein; CMS: chronic mild stress; SD: Sprague-Dawley; NF_Ϗ_β: Nuclear factor-_Ϗ_β MCAO: middle cerebral artery occlusion; OGD: Oxygen glucose deprivation.

**Table 4:** List of Nutraceutical Compounds targeting Inflammasomes in Cancer.

Nutraceutical Compound	Classification/Source	Overall Role in Inflammasomes	Experimental Model	Molecular Mechanism	Ref.
ECGC	Phenol/Green tea	NLRP3 inhibitor	-HMC	1. Inhibitory effect on proliferation 2. Suppresses NF_Ϗ_β activity 3. Decreases IL-1β secretion 4. Decreases caspase-1 activation	[128]
Luteoloside	*Taraxacum officinale* (L.) Weber ex F.H.Wigg. and *Cynara scolymus* L.	NLRP3 inhibitor	-HCC	1. Inhibition of cell migration and invasion 2. Suppresses proliferation and metastasis 3. Downregulates the expression level of caspase 1 and IL-1β	[132]
Isorhamnetin	Flavonoid/*Hippophae rhamnoides* L.	NLRP3, AIM2 inhibitor	-BMDMs	1. Downregulates the expression of pro-inflammatory cytokines 2. Attenuates the secretion of IL-1β resulting from NLRP3, NLRC4, and AIM2 inflammasome activation	[133]
Curcumin	Polyphenol/*Curcuma longa* L.	NLRP3 activator	-Malignant mesothelioma cells	1. Activates NLRP3 inflammasome 2. Activates the expression of caspase-1 3. Attenuates the expression of NF_Ϗ_β, TLR and IL-1β	[134]
Berberine	Alkaloid/Chinese herbs	NLRP3	-Triple-negative breast MDA-MB-231 cancer cells	1. Reduces pro-caspase-1, caspase-1, IL-1β, P2X7 and ASC expression	[135]
Polyphyllin VI	Saponin/*Trillium tschonoskii* Maxim.	NLRP3 activator	-Non-Small-Cell Lung A549 and H1299 cancer cells	1. Activation of caspase-1 via the induction of the ROS/NF_Ϗ_β /NLRP3/GSDMD signal axis 2. Upregulates NLRP3 inflammasome	[136]
Huaier extract	A kind of fungus/*Trametes robiniophila* Murr.	NLRP3 activator	-Non-Small-Cell Lung H520 -H358 cancer cells	1. Upregulates NLRP3 2. Activation of caspase-1, IL-1β, and IL-18	[137]
Anthocyanins	Natural pigment widely found in colored plants	NLRP3 activator	-Oral squamous HaCaT, Tca8113 -SCC15 cancer cells	1. Upregulates NLRP3 2. Activation of caspase-1 and IL-1β	[138]
DHA	ω-3FAs/Fish, Crustaceans, Molluscs, Eggs	NLRP3	-Myeloid-derived suppressor cells	1. Reduction in IL-1β secretion, inhibition of JNK pathway through β-arrestin-2 activation	[139]

HMC: Human melanoma cells; EGCG: Epigallocatechin-3-gallate; HCC: Hepatocellular carcinoma cells; BMDMs: Bone marrow-derived macrophages; NF_Ϗ_β: Nuclear factor-_Ϗ_β.

## Abbreviations

IS	immune system
ASC	apoptosis-associated speck-like protein
PAMPs	pathogen-associated molecular patterns
DAMPs	damage-associated molecular patterns
PRRs	pattern recognition receptors
TLRs	toll-like receptors
CLRs	c-type lectin receptors
RLRs	retinoic acid inducible gene-I (RIG1)-like receptors
OLRs	olygoadenylate synthetase-like receptors
CARD	caspase recruitment
PYR	pyrin domains
ROS	Reactive Oxygen Species
CVDs	cardiovascular diseases
PUFAs	polyunsaturated fatty acids

## References

[1] Schroder K., Tschopp J. (2010). The inflammasomes. Cell.

[2] Amin J., Boche D., Rakic S. (2017). What do we know about the inflammasome in humans?. Brain Pathol..

[3] Abderrazak A., Syrovets T., Couchie D., El Hadri K., Friguet B., Simmet T., Rouis M. (2015). NLRP3 inflammasome: From a danger signal sensor to a regulatory node of oxidative stress and inflammatory diseases. Redox Biol..

[4] Lamkanfi M., Dixit V.M. (2012). Inflammasomes and Their Roles in Health and Disease. Annu. Rev. Cell Dev. Biol..

[5] Kagan J.C., Barton G.M. (2014). Emerging principles governing signal transduction by pattern-recognition receptors. Cold Spring Harb. Perspect. Biol..

[6] Mogensen T.H. (2009). Pathogen recognition and inflammatory signaling in innate immune defenses. Clin. Microbiol. Rev..

[7] Uzman A. (2003). Molecular biology of the cell. Biochem. Mol. Biol. Educ..

[8] De Zoete M.R., Palm N.W., Zhu S., Flavell R.A. (2014). Inflammasomes. Cold Spring Harb. Perspect. Biol..

[9] Yaribeygi H., Mohammadi M.T., Rezaee R., Sahebkar A. (2018). Fenofibrate improves renal function by amelioration of NOX-4, IL-18, and p53 expression in an experimental model of diabetic nephropathy. J. Cell. Biochem..

[10] Kim H.Y., Lee H.J., Chang Y.J., Pichavant M., Shore S.A., Fitzgerald K.A., Iwakura Y., Israel E., Bolger K., Faul J. (2014). Interleukin-17-producing innate lymphoid cells and the NLRP3 inflammasome facilitate obesity-associated airway hyperreactivity. Nat. Med..

[11] Grebe A., Hoss F., Latz E. (2018). NLRP3 inflammasome and the IL-1 pathway in atherosclerosis. Circ. Res..

[12] Hoseini Z., Sepahvand F., Rashidi B., Sahebkar A., Masoudifar A., Mirzaei H. (2018). NLRP3 inflammasome: Its regulation and involvement in atherosclerosis. J. Cell. Physiol..

[13] Ference B.A., Ginsberg H.N., Graham I., Ray K.K., Packard C.J., Bruckert E., Hegele R.A., Krauss R.M., Raal F.J., Schunkert H. (2017). Low-density lipoproteins cause atherosclerotic cardiovascular disease. 1. Evidence from genetic, epidemiologic, and clinical studies. A consensus statement fromthe European Atherosclerosis Society Consensus Panel. Eur. Heart J..

[14] Zheng F., Xing S., Gong Z., Xing Q. (2013). NLRP3 inflammasomes show high expression in Aorta of patients with atherosclerosis. Heart Lung Circ..

[15] Ising C., Venegas C., Zhang S., Scheiblich H., Schmidt S.V., Vieira-Saecker A., Schwartz S., Albasset S., McManus R.M., Tejera D. (2019). NLRP3 inflammasome activation drives tau pathology. Nature.

[16] Heneka M.T., Kummer M.P., Stutz A., Delekate A., Schwartz S., Vieira-Saecker A., Griep A., Axt D., Remus A., Tzeng T.C. (2013). NLRP3 is activated in Alzheimer’s disease and contributes to pathology in APP/PS1 mice. Nature.

[17] Wang S., Yuan Y.H., Chen N.H., Wang H.B. (2019). The mechanisms of NLRP3 inflammasome/pyroptosis activation and their role in Parkinson’s disease. Int. Immunopharmacol..

[18] Moossavi M., Parsamanesh N., Bahrami A., Atkin S.L., Sahebkar A. (2018). Role of the NLRP3 inflammasome in cancer. Mol. Cancer.

[19] Liu L., Dong Y., Ye M., Jin S., Yang J., Joosse M.E., Sun Y., Zhang J., Lazarev M., Brant S.R. (2017). The Pathogenic Role of NLRP3 Inflammasome Activation in Inflammatory Bowel Diseases of Both Mice and Humans. J. Crohn’s Colitis.

[20] Mangan M.S.J., Olhava E.J., Roush W.R., Seidel H.M., Glick G.D., Latz E. (2018). Targeting the NLRP3 inflammasome in inflammatory diseases. Nat. Rev. Drug Discov..

[21] De Torre-Minguela C., del Castillo P.M., Pelegrín P. (2017). The NLRP3 and pyrin inflammasomes: Implications in the pathophysiology of autoinflammatory diseases. Front. Immunol..

[22] Zhang H., Li F., Li W.-W., Stary C., Clark J.D., Xu S., Xiong X. (2016). The inflammasome as a target for pain therapy. Br. J. Anaesth..

[23] Guo H., Callaway J.B., Ting J.P.-Y. (2015). Inflammasomes: Mechanism of action, role in disease, and therapeutics. Nat. Med..

[24] DeBusk R. (2009). Diet-Related Disease, Nutritional Genomics, and Food and Nutrition Professionals. J. Am. Diet. Assoc..

[25] Martín Ortega A.M., Segura Campos M.R. (2018). Bioactive Compounds as Therapeutic Alternatives. Bioactive Compounds: Health Benefits and Potential Applications.

[26] Madalena D.A., Pereira R.N., Vicente A.A., Ramos Ó.L. (2018). New insights on bio-based micro-and nanosystems in food. Encyclopedia of Food Chemistry.

[27] Mahfoudhi N., Ksouri R., Hamdi S. (2016). Nanoemulsions as potential delivery systems for bioactive compounds in food systems: Preparation, characterization, and applications in food industry. Emulsions.

[28] Gul K., Singh A.K., Jabeen R. (2016). Nutraceuticals and Functional Foods: The Foods for the Future World. Crit. Rev. Food Sci. Nutr..

[29] Pavillard L.E., Marín-Aguilar F., Bullon P., Cordero M.D. (2018). Cardiovascular diseases, NLRP3 inflammasome, and western dietary patterns. Pharmacol. Res..

[30] Lim H., Min D.S., Park H., Kim H.P. (2018). Flavonoids interfere with NLRP3 inflammasome activation. Toxicol. Appl. Pharmacol..

[31] Wu J., Luo Y., Jiang Q., Li S., Huang W., Xiang L., Liu D., Hu Y., Wang P., Lu X. (2019). Coptisine from Coptis chinensis blocks NLRP3 inflammasome activation by inhibiting caspase-1. Pharmacol. Res..

[32] Zhu T., Zhang L., Ling S., Duan J., Qian F., Li Y., Xu J.-W. (2014). Scropolioside B inhibits IL-1β and cytokines expression through NF-κB and inflammasome NLRP3 pathways. Mediat. Inflamm..

[33] Yan Y., Jiang W., Spinetti T., Tardivel A., Castillo R., Bourquin C., Guarda G., Tian Z., Tschopp J., Zhou R. (2013). Omega-3 Fatty Acids Prevent Inflammation and Metabolic Disorder through Inhibition of NLRP3 Inflammasome Activation. Immunity.

[34] Sun C., Wang X., Zheng G., Fan S., Lu J., Zhang Z., Wu D., Shan Q., Hu B., Zheng Y. (2016). Protective effect of different flavonoids against endothelial senescence via NLRP3 inflammasome. J. Funct. Foods.

[35] Ajdukovic J. (2015). The Role of NLRP3 Inflammasome in Cardiovascular Diseases. J. Clin. Exp. Cardiolog..

[36] Zhang X., Wang G., Gurley E.C., Zhou H. (2014). Flavonoid Apigenin Inhibits Lipopolysaccharide-Induced Inflammatory Response through Multiple Mechanisms in Macrophages. PLoS ONE.

[37] López-Franco O., Hernández-Vargas P., Ortiz-Muñoz G., Sanjuán G., Suzuki Y., Ortega L., Blanco J., Egido J., Gómez-Guerrero C. (2006). Parthenolide Modulates the NF-κB–Mediated Inflammatory Responses in Experimental Atherosclerosis. Arterioscler. Thromb. Vasc. Biol..

[38] Heinrich M., Robles M., West J.E., Ortiz De Montellano B.R., Rodriguez E. (1998). Ethnopharmacology of Mexican asteraceae (compositae). Annu. Rev. Pharmacol. Toxicol..

[39] Li S., Gao X., Wu X., Wu Z., Cheng L., Zhu L., Shen D., Tong X. (2015). Parthenolide inhibits LPS-induced inflammatory cytokines through the toll-like receptor 4 signal pathway in THP-1 cells. Acta Biochim. Biophys. Sin..

[40] Juliana C., Fernandes-Alnemri T., Wu J., Datta P., Solorzano L., Yu J.-W., Meng R., Quong A.A., Latz E., Scott C.P. (2010). Anti-inflammatory compounds parthenolide and Bay 11-7082 are direct inhibitors of the inflammasome. J. Biol. Chem..

[41] Kim J., Ahn H., Han B.-C., Lee S.-H., Cho Y.-W., Kim C.H., Hong E.-J., An B.-S., Jeung E.-B., Lee G.-S. (2014). Korean red ginseng extracts inhibit NLRP3 and AIM2 inflammasome activation. Immunol. Lett..

[42] Domiciano T.P., Wakita D., Jones H.D., Crother T.R., Verri W.A., Arditi M., Shimada K. (2017). Quercetin Inhibits Inflammasome Activation by Interfering with ASC Oligomerization and Prevents Interleukin-1 Mediated Mouse Vasculitis. Sci. Rep..

[43] Yu S.X., Du C.T., Chen W., Lei Q.Q., Li N., Qi S., Zhang X.J., Hu G.Q., Deng X.M., Han W.Y. (2015). Genipin inhibits NLRP3 and NLRC4 inflammasome activation via autophagy suppression. Sci. Rep..

[44] Zhang H., Chen X., Zong B., Yuan H., Wang Z., Wei Y., Wang X., Liu G., Zhang J., Li S. (2018). Gypenosides improve diabetic cardiomyopathy by inhibiting ROS-mediated NLRP3 inflammasome activation. J. Cell. Mol. Med..

[45] Li W., Chen M., Xu L., Lv Z., Chen L., Li Y., He W.F. (2019). Morroniside alleviates coxsackievirus B3-induced myocardial damage apoptosis via restraining NLRP3 inflammasome activation. RSC Adv..

[46] Ahn H., Lee G.S. (2017). Isorhamnetin and hyperoside derived from water dropwort inhibits inflammasome activation. Phytomedicine.

[47] Chalons P., Amor S., Courtaut F., Cantos-Villar E., Richard T., Auger C., Chabert P., Schni-Kerth V., Aires V., Delmas D. (2018). Study of potential anti-inflammatory effects of red wine extract and resveratrol through a modulation of interleukin-1-beta in macrophages. Nutrients.

[48] Deng Z.Y., Hu M.M., Xin Y.F., Gang C. (2015). Resveratrol alleviates vascular inflammatory injury by inhibiting inflammasome activation in rats with hypercholesterolemia and vitamin D2 treatment. Inflamm. Res..

[49] Dong W., Yang R., Yang J., Yang J., Ding J., Wu H., Zhang J. (2015). Resveratrol pretreatment protects rat hearts from ischemia/reperfusion injury partly via a NALP3 inflammasome pathway. Int. J. Clin. Exp. Pathol..

[50] Kong F., Ye B., Cao J., Cai X., Lin L., Huang S., Huang W., Huang Z. (2016). Curcumin Represses NLRP3 Inflammasome Activation via TLR4/MyD88/NF-κB and P2X7R Signaling in PMA-Induced Macrophages. Front. Pharmacol..

[51] Sun X., Wu A., Kwan Law B.Y., Liu C., Zeng W., Ling Qiu A.C., Han Y., He Y., Wai Wong V.K. (2020). The active components derived from Penthorum chinense Pursh protect against oxidative-stress-induced vascular injury via autophagy induction. Free Radic. Biol. Med..

[52] Qiu H., Liu W., Lan T., Pan W., Chen X., Wu H., Xu D. (2018). Salvianolate reduces atrial fibrillation through suppressing atrial interstitial fibrosis by inhibiting TGF-β1/Smad2/3 and TXNIP/NLRP3 inflammasome signaling pathways in post-MI rats. Phytomedicine.

[53] Li Y., Yang J., Chen M.-H., Wang Q., Qin M.-J., Zhang T., Chen X.-Q., Liu B.-L., Wen X.-D. (2015). Ilexgenin A inhibits endoplasmic reticulum stress and ameliorates endothelial dysfunction via suppression of TXNIP/NLRP3 inflammasome activation in an AMPK dependent manner. Pharmacol. Res..

[54] Jang S., Jeong S., Kim K.J., Kim H.J., Yu H.H., Park R., Kim H.M., You Y.O. (2003). Tanshinone IIA from Salvia miltiorrhiza Inhibits Inducible Nitric Oxide Synthase Expression and Production of TNF-α, IL-1β and IL-6 in Activated RAW 264.7 Cells. Planta Med..

[55] Hu Q., Wei B., Wei L., Hua K., Yu X., Li H., Ji H. (2015). Sodium tanshinone IIA sulfonate ameliorates ischemia-induced myocardial inflammation and lipid accumulation in Beagle dogs through NLRP3 inflammasome. Int. J. Cardiol..

[56] Hu Q., Zhang T., Yi L., Zhou X., Mi M. (2018). Dihydromyricetin inhibits NLRP3 inflammasome-dependent pyroptosis by activating the Nrf2 signaling pathway in vascular endothelial cells. BioFactors.

[57] Zhang B.C., Li Z., Xu W., Xiang C.H., Ma Y.F. (2018). Luteolin alleviates NLRP3 inflammasome activation and directs macrophage polarization in lipopolysaccharide-stimulated RAW264.7 cells. Am. J. Transl. Res..

[58] Martínez G.J., Robertson S., Barraclough J., Xia Q., Mallat Z., Bursill C., Celermajer D.S., Patel S. (2015). Colchicine Acutely Suppresses Local Cardiac Production of Inflammatory Cytokines in Patients With an Acute Coronary Syndrome. J. Am. Heart Assoc..

[59] Li R., Lu K., Wang Y., Chen M., Zhang F., Shen H., Yao D., Gong K., Zhang Z. (2017). Triptolide attenuates pressure overload-induced myocardial remodeling in mice via the inhibition of NLRP3 inflammasome expression. Biochem. Biophys. Res. Commun..

[60] Lv D., Cheng X., Tang L., Jiang M. (2017). The cardioprotective effect of total flavonoids on myocardial ischemia/reperfusion in rats. BioMed Pharmacother..

[61] Luo H., Fan Z., Xiang D., Jiang Z., Zhang W., Gao L., Feng C. (2018). The protective effect of umbelliferone ameliorates myocardial injury following ischemia-reperfusion in the rat through suppression NLRP3 inflammasome and upregulating the PPAR-γ. Mol. Med. Rep..

[62] Viljoen A., Mncwangi N., Vermaak I. (2012). Anti-Inflammatory Iridoids of Botanical Origin. Curr. Med. Chem..

[63] Badorff C., Fichtlscherer B., Rhoads R.E., Zeiher A.M., Muelsch A., Dimmeler S., Knowlton K.U. (2000). Nitric oxide inhibits dystrophin proteolysis by coxsackieviral protease 2A through S-nitrosylation: A protective mechanism against enteroviral cardiomyopathy. Circulation.

[64] Fairweather D.L., Rose N.R. (2007). Coxsackievirus-induced myocarditis in mice: A model of autoimmune disease for studying immunotoxicity. Methods.

[65] Kawaguchi M., Takahashi M., Hata T., Kashima Y., Usui F., Morimoto H., Izawa A., Takahashi Y., Masumoto J., Koyama J. (2011). Inflammasome Activation of Cardiac Fibroblasts Is Essential for Myocardial Ischemia/Reperfusion Injury. Circulation.

[66] Fu G.X., Li J.M., Zhou Y., Zhao S.P. (2007). Anti-inflammatory and immune suppressive effects of Conus officinalis glucosides in rats. Chin. J. Microbiol. Immunol..

[67] Sung Y.-H., Chang H.-K., Kim S.-E., Kim Y.-M., Seo J.-H., Shin M.-C., Shin M.-S., Yi J.-W., Shin D.-H., Kim H. (2009). Anti-Inflammatory and Analgesic Effects of the Aqueous Extract of Corni Fructus in Murine RAW 264.7 Macrophage Cells. J. Med. Food.

[68] Swanson D., Block R., Mousa S.A. (2012). Omega-3 Fatty Acids EPA and DHA: Health Benefits Throughout Life. Adv. Nutr..

[69] Sun C., Fan S., Wang X., Lu J., Zhang Z., Wu D., Shan Q., Zheng Y. (2015). Purple sweet potato color inhibits endothelial premature senescence by blocking the NLRP3 inflammasome. J. Nutr. Biochem..

[70] Calgarotto A.K., Maso V., Junior G.C.F., Nowill A.E., Filho P.L., Vassallo J., Saad S.T.O. (2018). Antitumor activities of Quercetin and Green Tea in xenografts of human leukemia HL60 cells. Sci. Rep..

[71] Wang C., Pan Y., Zhang Q.-Y., Wang F.-M., Kong L.-D. (2012). Quercetin and Allopurinol Ameliorate Kidney Injury in STZ-Treated Rats with Regulation of Renal NLRP3 Inflammasome Activation and Lipid Accumulation. PLoS ONE.

[72] Mozaffarian D., Benjamin E.J., Go A.S., Arnett D.K., Blaha M.J., Cushman M., Das S.R., De Ferranti S., Després J.P., Fullerton H.J. (2016). Heart disease and stroke statistics-2016 update a report from the American Heart Association. Circulation.

[73] Shishodia S. (2013). Molecular mechanisms of curcumin action: Gene expression. BioFactors.

[74] Zhang T., Chen Y.-M., Zhang G.-L. (2007). Novel Neolignan from Penthorum chinense. J. Integr. Plant. Biol..

[75] Wang M., Jiang Y., Liu H.L., Chen X.Q., Wu X., Zhang D.Y. (2014). A new flavanone from the aerial parts of Penthorum chinense. Nat. Prod. Res..

[76] Kirii H., Niwa T., Yamada Y., Wada H., Saito K., Iwakura Y., Asano M., Moriwaki H., Seishima M. (2003). Lack of Interleukin-1β Decreases the Severity of Atherosclerosis in ApoE-Deficient Mice. Arterioscler. Thromb. Vasc. Biol..

[77] Razani B., Feng C., Coleman T., Emanuel R., Wen H., Hwang S., Ting J.P., Virgin H.W., Kastan M.B., Semenkovich C.F. (2012). Autophagy links inflammasomes to atherosclerotic progression. Cell Metab..

[78] Ding C., Zhao Y., Shi X., Zhang N., Zu G., Li Z., Zhou J., Gao D., Lv L., Tian X. (2016). New insights into salvianolic acid A action: Regulation of the TXNIP/NLRP3 and TXNIP/ChREBP pathways ameliorates HFD-induced NAFLD in rats. Sci. Rep..

[79] Chang Y.P., Zhang H., Xie Y.M., Zeng X.B., Hu J., Zhuang Y. (2013). Analysis of salvianolate injection combined with usual drugs in treatment of coronary heart disease in real world. Zhongguo Zhongyao Zazhi.

[80] Williams B., Mancia G., Spiering W., Agabiti Rosei E., Azizi M., Buriner M., Clement D.L., Coca A., De Simone G., Dominiczak A. (2018). 2018 ESC/ESH Guidelines for the management of arterial hypertension|European Heart Journal|Oxford Academic. Eur. Heart J..

[81] Zhuang S., Cheng T.H., Shih N.L., Liu J.C., Chen J.J., Hong H.J., Chan P. (2016). Tanshinone IIA Induces Heme Oxygenase 1 Expression and Inhibits Cyclic Strain-Induced Interleukin 8 Expression in Vascular Endothelial Cells. Am. J. Chin. Med..

[82] Park J.C., Yu Y.B., Lee J.H. (1993). Isolation of steroids and flavonoids from the herb of Oenanthe javanica Dc. Korean J. Pharmacogn..

[83] Ji G., Yao X., Zang Z., Huang Z. (1990). Antiarrhythmic effect of Oenanthe javanica (Bl.) DC. injection. Zhongguo Zhong Yao Za Zhi.

[84] Xin-Bo Y., Zheng-Ming H., Wen-Bin C., Ming Z., Hong-Yan C., Jing-Zhen Z. (2000). Antidiabetic effect of Oenanthe javanica flavone. Acta Pharmacol. Sin..

[85] Ku S.K., Kim T.H., Lee S., Kim S.M., Bae J.S. (2013). Antithrombotic and profibrinolytic activities of isorhamnetin-3-O-galactoside and hyperoside. Food Chem. Toxicol..

[86] Goldberg D.M., Soleas G.J., Levesque M. (1999). Moderate alcohol consumption: The gentle face of janus. Clin. Biochem..

[87] Hollingsworth P.M., Forrest L.L., Spouge J.L., Hajibabaei M., Ratnasingham S., van der Bank M., Chase M.W., Cowan R.S., Erickson D.L., Fazekas A.J. (2009). A DNA barcode for land plants. Proc. Natl. Acad. Sci. USA.

[88] Bonnefont-Rousselot D. (2016). Resveratrol and cardiovascular diseases. Nutrients.

[89] Cui W.X., Yang J., Chen X.Q., Mao Q., Wei X.L., Wen X.D., Wang Q. (2013). Triterpenoid-rich fraction from ilex hainanensis merr. attenuates non-alcoholic fatty liver disease induced by high fat diet in rats. Am. J. Chin. Med..

[90] Qiu Y., Tang L. (2016). Roles of the NLRP3 inflammasome in the pathogenesis of diabetic nephropathy. Pharmacol. Res..

[91] Ding X.-Q., Wu W.-Y., Jiao R.-Q., Gu T.-T., Xu Q., Pan Y., Kong L.-D. (2018). Curcumin and allopurinol ameliorate fructose-induced hepatic inflammation in rats via miR-200a-mediated TXNIP/NLRP3 inflammasome inhibition. Pharmacol. Res..

[92] Chen W., Wang J., Luo Y., Wang T., Li X., Li A., Li J., Liu K., Liu B. (2016). Ginsenoside Rb1 and compound K improve insulin signaling and inhibit ER stress-associated NLRP3 inflammasome activation in adipose tissue. J. Ginseng Res..

[93] Ahsan H., Ahad A., Iqbal J., Siddiqui W.A. (2014). Pharmacological potential of tocotrienols: A review. Nutr. Metab..

[94] Samra Y.A., Said H.S., Elsherbiny N.M., Liou G.I., El-Shishtawy M.M., Eissa L.A. (2016). Cepharanthine and Piperine ameliorate diabetic nephropathy in rats: Role of NF-κB and NLRP3 inflammasome. Life Sci..

[95] Lu M., Yin N., Liu W., Cui X., Chen S., Wang E. (2017). Curcumin Ameliorates Diabetic Nephropathy by Suppressing NLRP3 Inflammasome Signaling. BioMed Res. Int..

[96] Abderrazak A., El Hadri K., Bosc E., Blondeau B., Slimane M.-N., Buchele B., Simmet T., Couchie D., Rouis M. (2016). Inhibition of the Inflammasome NLRP3 by Arglabin Attenuates Inflammation, Protects Pancreatic β-Cells from Apoptosis, and Prevents Type 2 Diabetes Mellitus Development in ApoE2Ki Mice on a Chronic High-Fat Diet. J. Pharmacol. Exp. Ther..

[97] Li A., Zhang S., Li J., Liu K., Huang F., Liu B. (2016). Metformin and resveratrol inhibit Drp1-mediated mitochondrial fission and prevent ER stress-associated NLRP3 inflammasome activation in the adipose tissue of diabetic mice. Mol. Cell. Endocrinol..

[98] Lu L., Lu Q., Chen W., Li J., Li C., Zheng Z. (2018). Vitamin D 3 Protects against Diabetic Retinopathy by Inhibiting High-Glucose-Induced Activation of the ROS/TXNIP/NLRP3 Inflammasome Pathway. J. Diabetes Res..

[99] Xu X., Chen Y., Song J., Hou F., Ma X., Liu B., Huang F. (2018). Mangiferin suppresses endoplasmic reticulum stress in perivascular adipose tissue and prevents insulin resistance in the endothelium. Eur. J. Nutr..

[100] Ma Q., Yang Q., Chen J., Yu C., Zhang L., Zhou W., Chen M. (2020). Salvianolic Acid A Ameliorates Early-Stage Atherosclerosis Development by Inhibiting NLRP3 Inflammasome Activation in Zucker Diabetic Fatty Rats. Molecules.

[101] Lalitha N., Sadashivaiah B., Ramaprasad T.R., Singh S.A. (2020). Anti-hyperglycemic activity of myricetin, through inhibition of DPP-4 and enhanced GLP-1 levels, is attenuated by co-ingestion with lectin-rich protein. PLoS ONE.

[102] Zhu M.-J., Kang Y., Xue Y., Liang X., García M.P.G., Rodgers D., Kagel D.R., Du M. (2018). Red raspberries suppress NLRP3 inflammasome and attenuate metabolic abnormalities in diet-induced obese mice. J. Nutr. Biochem..

[103] Zhu Y., Zhu C., Yang H., Deng J., Fan D. (2020). Protective effect of ginsenoside Rg5 against kidney injury via inhibition of NLRP3 inflammasome activation and the MAPK signaling pathway in high-fat diet/streptozotocin-induced diabetic mice. Pharmacol. Res..

[104] Eo H., Lee H.-J., Lim Y. (2016). Ameliorative effect of dietary genistein on diabetes induced hyper-inflammation and oxidative stress during early stage of wound healing in alloxan induced diabetic mice. Biochem. Biophys. Res. Commun..

[105] Wang W., Wang C., Ding X.-Q., Pan Y., Gu T.-T., Wang M.-X., Liu Y.-L., Wang F.-M., Wang S.-J., Kong L.-D. (2013). Quercetin and allopurinol reduce liver thioredoxin-interacting protein to alleviate inflammation and lipid accumulation in diabetic rats. Br. J. Pharmacol..

[106] Harbis A., Perdreau S., Vincent-Baudry S., Charbonnier M., Bernard M.-C., Raccah D., Senft M., Lorec A.-M., Defoort C., Portugal H. (2004). Glycemic and insulinemic meal responses modulate postprandial hepatic and intestinal lipoprotein accumulation in obese, insulin-resistant subjects. Am. J. Clin. Nutr..

[107] Shah S., Shah G., Singh S., Gohil P., Chauhan K., Shah K., Chorawala M. (2011). Effect of piperine in the regulation of obesity-induced dyslipidemia in high-fat diet rats. Indian J. Pharmacol..

[108] Cao C., Su M., Zhou F. (2017). Mangiferin inhibits hippocampal NLRP3 inflammasome and exerts antidepressant effects in a chronic mild stress mice model. Behav. Pharmacol..

[109] Alcocer-Gómez E., Casas-Barquero N., Williams M.R., Romero-Guillena S.L., Cañadas-Lozano D., Bullón P., Sánchez-Alcazar J.A., Navarro-Pando J.M., Cordero M.D. (2017). Antidepressants induce autophagy dependent-NLRP3-inflammasome inhibition in Major depressive disorder. Pharmacol. Res..

[110] Li R., Wang X., Qin T., Qu R., Ma S. (2016). Apigenin ameliorates chronic mild stress-induced depressive behavior by inhibiting interleukin-1β production and NLRP3 inflammasome activation in the rat brain. Behav. Brain Res..

[111] Sui D., Xie Q., Yi W., Gupta S., Yu X., Li J., Wang J., Wang J., Deng X. (2016). Resveratrol Protects against Sepsis-Associated Encephalopathy and Inhibits the NLRP3/IL-1 *β* Axis in Microglia. Mediat. Inflamm..

[112] Wang X., Li R., Wang X., Fu Q., Ma S. (2015). Umbelliferone ameliorates cerebral ischemia–reperfusion injury via upregulating the PPAR gamma expression and suppressing TXNIP/NLRP3 inflammasome. Neurosci. Lett..

[113] Yu C., He Q., Zheng J., Li L.Y., Hou Y.H., Song F.Z. (2017). Sulforaphane improves outcomes and slows cerebral ischemic/reperfusion injury via inhibition of NLRP3 inflammasome activation in rats. Int. Immunopharmacol..

[114] Yang F., Wang Z., Wei X., Han H., Meng X., Zhang Y., Shi W., Li F., Xin T., Pang Q. (2014). NLRP3 deficiency ameliorates neurovascular damage in experimental ischemic stroke. J. Cereb. Blood Flow Metab..

[115] Li M., Li H., Fang F., Deng X., Ma S. (2017). Astragaloside IV attenuates cognitive impairments induced by transient cerebral ischemia and reperfusion in mice via anti-inflammatory mechanisms. Neurosci. Lett..

[116] Cao G., Jiang N., Hu Y., Zhang Y., Wang G., Yin M., Ma X., Zhou K., Qi J., Yu B. (2016). Ruscogenin attenuates cerebral ischemia-induced blood-brain barrier dysfunction by suppressing TXNIP/NLRP3 inflammasome activation and the MAPK pathway. Int. J. Mol. Sci..

[117] Qiu J., Wang M., Zhang J., Cai Q., Lu D., Li Y., Dong Y., Zhao T., Chen H. (2016). The neuroprotection of Sinomenine against ischemic stroke in mice by suppressing NLRP3 inflammasome via AMPK signaling. Int. Immunopharmacol..

[118] Zhang S., Jiang L., Che F., Lu Y., Xie Z., Wang H. (2017). Arctigenin attenuates ischemic stroke via SIRT1-dependent inhibition of NLRP3 inflammasome. Biochem. Biophys. Res. Commun..

[119] He Q., Li Z., Wang Y., Hou Y., Li L., Zhao J. (2017). Resveratrol alleviates cerebral ischemia/reperfusion injury in rats by inhibiting NLRP3 inflammasome activation through Sirt1-dependent autophagy induction. Int. Immunopharmacol..

[120] Li Y., Li J., Li S., Li Y., Wang X., Liu B., Fu Q., Ma S. (2015). Curcumin attenuates glutamate neurotoxicity in the hippocampus by suppression of ER stress-associated TXNIP/NLRP3 inflammasome activation in a manner dependent on AMPK. Toxicol. Appl. Pharmacol..

[121] Zhang N., Zhang X., Liu X., Wang H., Xue J., Yu J., Kang N., Wang X. (2014). Chrysophanol Inhibits NALP3 Inflammasome Activation and Ameliorates Cerebral Ischemia/Reperfusion in Mice. Mediat. Inflamm..

[122] Zhang Y., Hu W., Zhang B., Yin Y., Zhang J., Huang D., Huang R., Li W., Li W. (2017). Ginsenoside Rg1 protects against neuronal degeneration induced by chronic dexamethasone treatment by inhibiting NLRP-1 inflammasomes in mice. Int. J. Mol. Med..

[123] Che H., Li Q., Zhang T., Wang D., Yang L., Xu J., Yanagita T., Xue C., Chang Y., Wang Y. (2018). Effects of Astaxanthin and Docosahexaenoic-Acid-Acylated Astaxanthin on Alzheimer’s Disease in APP/PS1 Double-Transgenic Mice. J. Agric. Food Chem..

[124] Fraga V.G., das Graças Carvalho M., Caramelli P., de Sousa L.P., Gomes K.B. (2017). Resolution of inflammation, n-3 fatty acid supplementation and Alzheimer disease: A narrative review. J. Neuroimmunol..

[125] Chow M.T., Sceneay J., Paget C., Wong C.S.F., Duret H., Tschopp J., Moller A., Smyth M.J. (2012). NLRP3 Suppresses NK Cell-Mediated Responses to Carcinogen-Induced Tumors and Metastases. Cancer Res..

[126] Cătană C.-S., Atanasov A.G., Berindan-Neagoe I. (2018). Natural products with anti-aging potential: Affected targets and molecular mechanisms. Biotechnol. Adv..

[127] Jhang J.-J., Lin J.-H., Yen G.-C. (2018). Beneficial Properties of Phytochemicals on NLRP3 Inflammasome-Mediated Gout and Complication. J. Agric. Food Chem..

[128] Ellis L.Z., Liu W., Luo Y., Okamoto M., Qu D., Dunn J.H., Fujita M. (2011). Green tea polyphenol epigallocatechin-3-gallate suppresses melanoma growth by inhibiting inflammasome and IL-1β secretion. Biochem. Biophys. Res. Commun..

[129] Shin H.-Y., Kim S.-H., Jeong H.-J., Kim S.-Y., Shin T.-Y., Um J.-Y., Hong S.-H., Kim H.-M. (2007). Epigallocatechin-3-Gallate Inhibits Secretion of TNF-α, IL-6 and IL-8 through the Attenuation of ERK and NF-κB in HMC-1 Cells. Int. Arch. Allergy Immunol..

[130] Tsai P.-Y., Ka S.-M., Chang J.-M., Chen H.-C., Shui H.-A., Li C.-Y., Hua K.-F., Chang W.-L., Huang J.-J., Yang S.-S. (2011). Epigallocatechin-3-gallate prevents lupus nephritis development in mice via enhancing the Nrf2 antioxidant pathway and inhibiting NLRP3 inflammasome activation. Free Radic. Biol. Med..

[131] Umeda D., Yano S., Yamada K., Tachibana H. (2008). Green Tea Polyphenol Epigallocatechin-3-gallate Signaling Pathway through 67-kDa Laminin Receptor. J. Biol. Chem..

[132] Fan S., Wang Y., Lu J., Zheng Y., Wu D., Li M., Hu B., Zhang Z., Cheng W., Shan Q. (2014). Luteoloside Suppresses Proliferation and Metastasis of Hepatocellular Carcinoma Cells by Inhibition of NLRP3 Inflammasome. PLoS ONE.

[133] Lu X., Liu T., Chen K., Xia Y., Dai W., Xu S., Xu L., Wang F., Wu L., Li J. (2018). Isorhamnetin: A hepatoprotective flavonoid inhibits apoptosis and autophagy via P38/PPAR-α pathway in mice. BioMed Pharmacother..

[134] Miller J.M., Thompson J.K., MacPherson M.B., Beuschel S.L., Westbom C.M., Sayan M., Shukla A. (2014). Curcumin: A Double Hit on Malignant Mesothelioma. Cancer Prev. Res..

[135] Yao M., Fan X., Yuan B., Takagi N., Liu S., Han X., Ren J., Liu J. (2019). Berberine inhibits NLRP3 Inflammasome pathway in human triple-negative breast cancer MDA-MB-231 cell. BMC Complement. Altern. Med..

[136] Teng J.-F., Mei Q.-B., Zhou X.-G., Tang Y., Xiong R., Qiu W.-Q., Pan R., Law B.Y.-K., Wong V.K.-W., Yu C.-L. (2020). Polyphyllin VI Induces Caspase-1-Mediated Pyroptosis via the Induction of ROS/NF-κB/NLRP3/GSDMD Signal Axis in Non-Small Cell Lung Cancer. Cancers.

[137] Xie J., Zhuan B., Wang H., Wang Y., Wang X., Yuan Q., Yang Z. (2019). Huaier extract suppresses non-small cell lung cancer progression through activating NLRP3-dependent pyroptosis. Anat. Rec..

[138] Yue E., Tuguzbaeva G., Chen X., Qin Y., Li A., Sun X., Dong C., Liu Y., Yu Y., Zahra S.M. (2019). Anthocyanin is involved in the activation of pyroptosis in oral squamous cell carcinoma. Phytomedicine.

[139] Dumont A., de Rosny C., Kieu T.-L.-V., Perrey S., Berger H., Fluckiger A., Muller T., Pais de Barros J.-P., Pichon L., Hichami A. (2019). Docosahexaenoic acid inhibits both NLRP3 inflammasome assembly and JNK-mediated mature IL-1β secretion in 5-fluorouracil-treated MDSC: Implication in cancer treatment. Cell Death Dis..

[140] Yuldashev M.P. (1997). Cynaroside content of the plants Ferula varia and F. foetida. Chem. Nat. Compd..

[141] Hu C., Kitts D.D. (2004). Luteolin and luteolin-7-O-glucoside from dandelion flower suppress iNOS and COX-2 in RAW264.7 cells. Mol. Cell. Biochem..

[142] Sun X., Sun G., Wang M., Xiao J., Sun X. (2011). Protective effects of cynaroside against H2O2-induced apoptosis in H9c2 cardiomyoblasts. J. Cell. Biochem..

[143] Xiong J., Li S., Wang W., Hong Y., Tang K., Luo Q. (2013). Screening and identification of the antibacterial bioactive compounds from Lonicera japonica Thunb. leaves. Food Chem..

